# Locoregional Immune Checkpoint Blockade and Remodeling of Lymph Nodes by Engineered Dendritic Cell‐Derived Exosomes for Suppressing Tumor Progression and Metastasis

**DOI:** 10.1002/advs.202500139

**Published:** 2025-04-03

**Authors:** Yizhen Wang, Xiaomin Guo, Jingya Qin, Yifan Xue, Peng Zhang, Yadong Liu, Moyang Chen, Guanghao Zhu, Xinqiu Song, Lili Cheng, Bo Liu, Jie Liu, Jie Ren

**Affiliations:** ^1^ Department of Ultrasound The Third Affiliated Hospital of Sun Yat‐sen University Guangzhou 510630 P. R. China; ^2^ School of Biomedical Engineering Sun Yat‐sen University Shenzhen 518107 P. R. China; ^3^ Department of Hepatobiliary Pancreatic Splenic Surgery The Third Affiliated Hospital of Sun Yat‐sen University Guangzhou 510630 P. R. China

**Keywords:** engineered exosomes, immune microenvironment, local immunotherapy, metastasis, tumor‐draining lymph nodes

## Abstract

Tumor‐draining lymph nodes (TDLNs) are the primary sites of eliciting anti‐tumor immunity, which play an important role in controlling tumor progression and metastasis. However, the immunosuppressive microenvironment of TDLNs propels the formation of pre‐metastatic niche, in which the immunocytes are dysfunctional, and the high expression of programmed death‐ligand 1 (PD‐L1) on dendritic cells (DCs) restricts the activation of cytotoxic T lymphocytes. Herein, engineered exosomes (EmDEX@GA) are developed for locoregional immunomodulation of TDLNs. EmDEX@GA possess CC‐chemokine receptor 7 (CCR7) ‐dependent LN homing capacity and over‐expressed programmed cell death protein 1 (PD‐1) for immune checkpoint blockade (ICB). The loaded stimulator of interferon genes (STING) agonist can reinforce anti‐tumor immunity through STING pathway activation. In orthotopic breast cancer mouse model, local administration of EmDEX@GA remodels the immunosuppressive microenvironment of TDLNs and elicits potent anti‐tumor immunity, resulting in the suppression of tumor as well as the reduction of lymph node metastasis and distant metastasis. Compared with systemic ICB, local immunotherapy with EmDEX@GA has better therapeutic efficacy on suppressing distant metastasis. Moreover, the study suggests that the occurrences of distant metastasis are associated with the immunosuppressive microenvironment rather than the metastasis in TDLNs, indicating that targeted immunomodulation of TDLNs is necessary.

## Graphical abstract

The study develops engineered exosomes (EmDEX@GA) for locoregional immunomodulation of tumor‐draining lymph node (TDLN). EmDEX@GA can actively target to TDLN and remodel its immunosuppressive microenvironment through the synergistic effects of PD‐1/PD‐L1 blockade and STING pathway activation, subsequently enhance anti‐tumor immunity and suppress tumor progression and metastasis. Compared with systemic immunotherapy, EmDEX@GA present better therapeutic efficacy on suppressing distant metastasis.

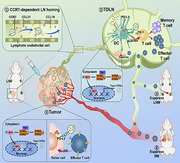

## 1 Introduction

As a typical route of metastasis, lymph node metastasis (LNM) often occurs in solid malignant tumor, which is regarded as an informative prognostic factor.^[^ ^1^ ^]^ Whether associated with distant metastasis (DM), the presence of LNM has important clinical significance on tumor progression and stage. It always represents poor prognosis and determines the choice of treatments.^[^ ^2^, ^3^ ^]^ Lymph nodes (LNs) are secondary lymphoid tissues of organisms. They serve as immune centers where antigen‐presenting cells (APCs) activate T cells and initiate a series of anti‐tumor immune responses.^[^ ^4^ ^]^ When an immune organ happens to be a site of metastasis, it will inevitably impair the function of LNs. Thus, inhibiting LNM is essential to control tumor progression, improve prognosis and prolong survival time. While finding the way to inhibit LNM, the mechanisms of LNM were widely studied and the crucial role of tumor‐draining lymph nodes (TDLNs) in tumor metastasis has been gradually recognized.^[^ ^5^ ^]^ Anatomically, TDLNs are the first place that APCs migrate into and present tumor antigens to naive T cells to elicit anti‐tumor immunity at an early time. Simultaneously, TDLNs are the nearest LNs that tumor cells could reach via lymphatic pathway. So technically, they are the crossroad of metastasis and immunity.^[^ ^6^, ^7^ ^]^ However, as the first line of defense, TDLNs exhibit regional immune suppression compared with non‐draining lymph nodes (NDLNs) or healthy controls.^[^ ^8^ ^]^ Tumor‐derived antigens, secreted factors and extracellular vesicles draining from afferent lymphatic vessel make changes on the structure and functions of TDLNs.^[^ ^9^ ^]^ They undergo expansion and stromal remodeling, as well as lymphangiogenesis and high endothelial venules (HEVs) dedifferentiation. At the same time, immunocytes in TDLNs are affected including dendritic cells (DCs) dysfunction, impaired effector T cells and accumulation of regulatory T cells (Tregs). These alterations progressively transform TDLNs into pre‐metastatic niche that supports the invasion, adhesion and seeding of tumor cells.^[^ ^8^, ^10^ ^]^ Furthermore, given that TDLNs are the primary locations of tumor‐associated antigens, the immunosuppressive status will impair anti‐tumor immunity and systemic immune surveillance, resulting in tumor progression and distant metastasis.^[^ ^11^ ^]^

The number and functions of DCs in the immunosuppressive TDLNs are commonly defective. However, studies found that the problems of DCs were not only immaturity. Immunosuppressive ligand programmed death‐ligand 1 (PD‐L1) was highly expressed on DCs in TDLNs from both patients and mouse model.^[^ ^12^, ^13^ ^]^ Meanwhile, specific blockade of DC‐derived PD‐L1 improved T cell activation and anti‐tumor immunity.^[^ ^12^, ^14^, ^15^, ^16^ ^]^ The binding of PD‐L1 and programmed cell death protein 1 (PD‐1) is usually known to take place in tumor microenvironment (TME) to induce immune evasion. But actually, the PD‐1/PD‐L1 interactions exist in LNs as well. Apart from tumor cells, myeloid cells like DCs and macrophages in TDLNs will express PD‐L1 that would restrict the priming and proliferation of T cells.^[^ ^17^, ^18^ ^]^ Growing evidences have revealed the vital role of TDLNs in immune checkpoint blockade (ICB) efficacy. Dammeijer et al.^[^ ^17^ ^]^ found that PD‐1/PD‐L1 interactions occurred in TDLNs but not in tumor correlated with prognosis of melanoma patients. Francis et al.^[^ ^19^ ^]^ suggested that targeted ICB administration to TDLNs achieved stronger anti‐tumor immunity and better therapeutic effects compared with systemic ICB therapy. All of these data indicate that TDLNs could be potential immunotherapeutic targets of ICB to potently enhance anti‐tumor immunity. In this case, we presume that locally conducting PD‐1/PD‐L1 blockade and remodeling the immune microenvironment of TDLNs could elicit sufficient anti‐tumor immunity to suppress tumor progression, LNM and DM. To demonstrate the perspective, we need to locally administrate immune checkpoint inhibitors (ICIs) to TDLNs and find out how exactly that remodeling the immune microenvironment of TDLNs will affect the progression and metastasis of tumor. However, the conventional systemic administration of ICIs may not be an appropriate method owing to the poor access and enrichment in TDLNs, which could explain the relatively limited response of the treatment to some extent.^[^ ^20^, ^21^ ^]^ Locoregional LN delivery of nanocarriers is promising because nanocarriers possess the fit size to drain through the channels (≈100 nm in diameter) of lymphatic capillaries and accumulate in LNs.^[^ ^22^, ^23^, ^24^ ^]^ Especially, nanocarriers like exosomes could be engineered to achieve enhanced LN targeting and diverse functions of immunomodulation.^[^ ^25^ ^]^ Exosomes are 30–150 nm extracellular vesicles produced by different kinds of cells.^[^ ^26^ ^]^ They have bilayer membrane structure and contain many biomolecules that could reflect their parental cells.^[^ ^27^, ^28^ ^]^ Exosomes derived from immunocytes like DCs could maintain the essential immunostimulatory capabilities and have great interactions within immunocytes, making them suitable for local immunomodulation in TDLNs.^[^ ^29^, ^30^ ^]^

**Figure 1:**
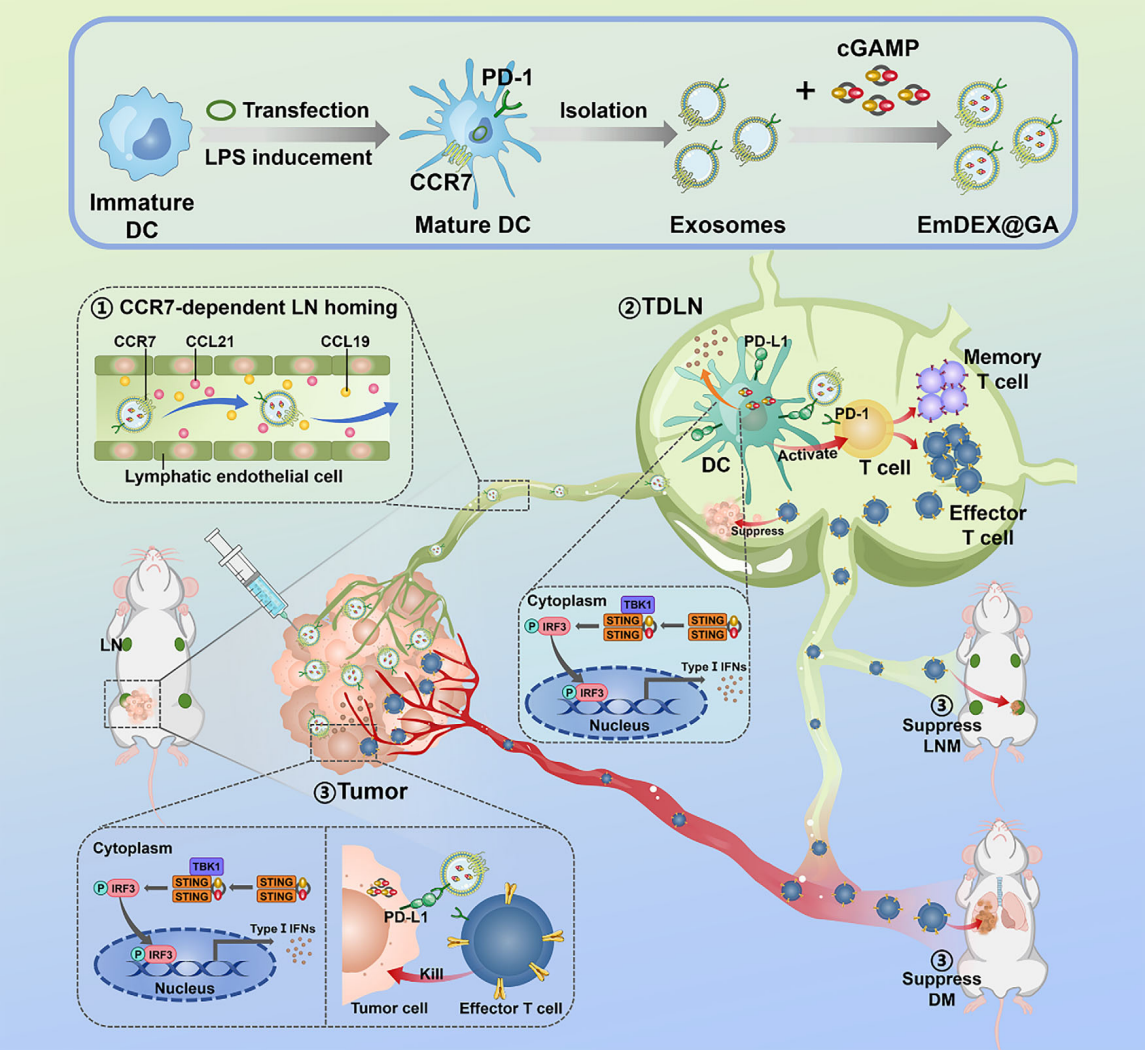
Schematic illustration of the preparation of EmDEX@GA and mechanisms of locoregional immune microenvironment remodeling for suppressing tumor progression and metastasis. 1) After intratumoral administration, EmDEX@GA actively target to tumor‐draining lymph node (TDLN) via CCR7‐dependent homing capacity. 2) In TDLN, EmDEX@GA perform PD‐1/PD‐L1 blockade and STING activation on dendritic cells (DCs), which can prime and activate T cells as well as promote the production of type I interferons (IFN). The activated effector T cells directly suppress the occurrence of metastasis inside TDLN. 3) The efferent effector T cells are recruited to tumor site and kill tumor cells with the help of STING‐mediated IFN signaling and PD‐L1 blockade conducted by the remaining EmDEX@GA. Besides, the efferent effector T cells can flow systemically to suppress the formation of lymph node metastasis (LNM) and distant metastasis (DM).

Herein, we designed engineered exosomes EmDEX@GA for locoregional PD‐1/PD‐L1 blockade and immune microenvironment remodeling in TDLNs to elicit systemic anti‐tumor immunity (**Figure** **1**). In consideration of the natural behavior of biology, we utilized exosomes of mature DCs as nanocarriers to achieve CC‐chemokine receptor 7 (CCR7) ‐dependent homing to LNs. CCR7 is the most important chemokine receptor for guiding immunocytes migrate to lymph nodes. Mature DCs highly express CCR7 that guiding the migration of them all the way to LNs through binding with CC‐chemokine ligand 19 (CCL19) and CCL21.^[^ ^31^, ^32^ ^]^ Additionally, we engineered the parental DCs to over‐express PD‐1. Thus, the isolated exosomes can naturally exhibit CCR7‐dependent active targeting to TDLNs and block DC‐derived PD‐L1. Ultimately, stimulator of interferon genes (STING) agonist, 2′,3′‐cyclic GMP‐AMP (cGAMP), was loaded inside the exosomes to form EmDEX@GA. STING pathway is an essential regulator of anti‐tumor immunity.^[^ ^33^ ^]^ The activation of STING pathway promotes the production of type I interferons (IFN‐α and IFN‐β), which are benefit for DC maturation and its ability of antigen presentation, as well as T cells recruitment and infiltration.^[^ ^34^, ^35^ ^]^ After constructing the expected engineered exosomes, we established the orthotopic mouse model of breast cancer that would occur LNM and DM over time. While intratumoral injection, EmDEX@GA achieved dual accumulation in TME and TDLNs, performed immune microenvironment remodeling via the synergistic effects of PD‐1/PD‐L1 blockade and STING‐mediated immunocytes activation, subsequently enhanced anti‐tumor immunity and further suppressed tumor progression and metastasis. Compared with systemic ICB therapy, the locoregional EmDEX@GA administration exhibited more effective suppression on LNM and DM with lower dosage, proved the availability of the local TDLN immunomodulation strategy. Besides, we analyzed the association between TDLN immunosuppressive microenvironment and distant metastasis, further confirmed the important role of TDLN immune microenvironment in controlling tumor progression and metastasis.

## 2 Results and Discussion

### 2.1 Preparation and Characterization of EmDEX@GA

**Figure 2:**
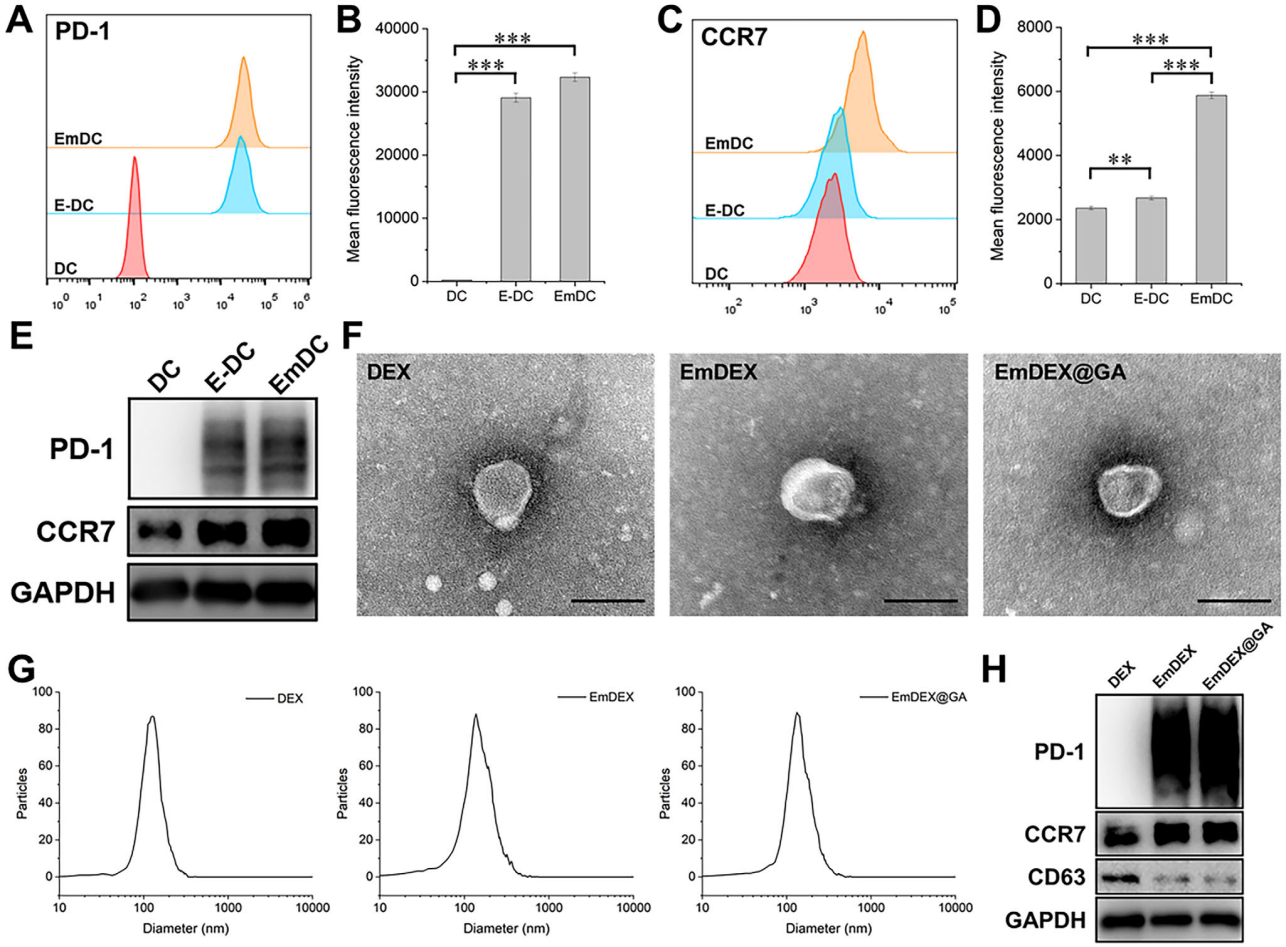
Preparation and characterization of EmDEX@GA. A) Flow cytometry analysis of PD‐1 expression on DCs and its B) mean fluorescence intensity (MFI) (*n* = 3, ***p < 0.001). C) Flow cytometry analysis of CCR7 expression on DCs and its D) MFI (*n* = 3, **p < 0.01, ***p < 0.001). E) WB analysis of the expression of PD‐1 and CCR7 on DCs. F) TEM images of different groups of exosomes (scale bar: 100 nm). G) Size distribution of exosomes measured by NTA. H) WB analysis of the protein markers of exosomes.

In normal immune activities, mature DCs migrate to LNs through chemotaxis mediated by highly expressed CCR7. Exosomes derived from mature DCs will retain this natural biologic behavior, thus becoming appropriate nanocarriers for local LN drug delivery. In this study, exosomes were isolated from the culture supernatant of mature DCs with engineered PD‐1 over‐expression. After that, cGAMP was loaded inside the exosomes. To perform the preparation process, the parental DCs with corresponding phenotype were constructed first. DC2.4 cells were genetically engineered by lentivirus transfection to over‐express PD‐1. Then, lipopolysaccharide (LPS) was utilized to induce the maturation of transfected DC2.4 cells. While the process of maturation, CCR7 expression would be upregulated simultaneously. The expression of PD‐1 and CCR7 on different phases of DCs were confirmed by flow cytometry and western blot (WB) analysis. DCs after lentivirus transfection (E‐DC) and the subsequent LPS inducement (EmDC) highly expressed PD‐1 as we designed (**Figure** **2**A,B). The 100% expression of PD‐1 indicated the success of transfection (Figure S1, Supporting Information). Figure S2 (Supporting Information) shows that the maturation percentage of EmDC was raised to above 70% after transfection and LPS inducement. Correspondingly, the expression of CCR7 was largely upregulated on EmDC for more than twofold compared with untreated DC (**Figure** **2**C,D). The results of WB showed the consistent alteration with flow cytometry (Figure 2E). After the successful construction of parental DCs, exosomes (EmDEX) were isolated and cGAMP was loaded to form EmDEX@GA. The average drug loading of cGAMP was 2.48% via calculation. Images of transmission electron microscopy (TEM) showed the morphology of exosomes (Figure 2F). EmDEX, EmDEX@GA together with normal DC exosomes (DEX) all appeared round membrane vesicles with diameter about 100 nm. Nanoparticle tracking analysis (NTA) was conducted to further confirm the size of exosomes (Figure 2G). Most of the exosomes had diameters ranged from 80 to 200 nm, and the peaks of all three types of exosomes were around 130 nm, which corresponded with our expectation. To test the stability of EmDEX@GA, we measured their size distribution after being preserved at 4 and 37 °C for 24 h. The diameters of EmDEX@GA maintained in a certain range, with peaks at 142 and 164 nm, respectively (Figure S3, Supporting Information). These data indicated the satisfactory stability of EmDEX@GA. Additionally, results of WB demonstrated that EmDEX and EmDEX@GA possessed the PD‐1 and CCR7 over‐expression identical with their parental cells (Figure 2H). The load of cGAMP did not affect the phenotype of exosomes. The PD‐1 expression percentage of EmDEX@GA was 66.2% (Figure S4A, Supporting Information). Compared with DEX, the expression percentage of CCR7 increased from 15.7% to 49.1% (Figure S4B, Supporting Information). With these characteristics, EmDEX@GA possessed the potential of CCR7‐dependent LN targeting, PD‐1/PD‐L1 blockade and STING pathway activation. Thus, making them suitable to be further utilized in vitro and in vivo.

### 2.2 In Vitro PD‐1/PD‐L1 Blockade and STING Activation of EmDEX@GA

**Figure 3:**
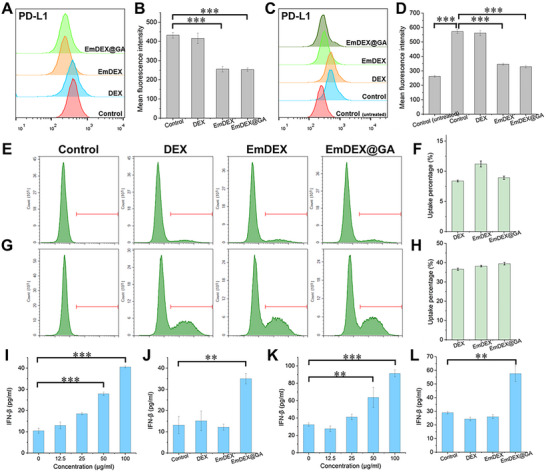
In vitro ICB and STING activation of EmDEX@GA. A) PD‐L1 detection on 4T1 cells treated with different groups of exosomes and its B) MFI by flow cytometry (*n* = 3, ***p < 0.001). C) PD‐L1 detection on DC2.4 cells untreated or treated with 4T1 culture medium and exosomes by flow cytometry and its D) MFI (*n* = 3, ***p < 0.001). E–H) Cellular uptake of different groups of exosomes by E, F) 4T1 cells and G, H) DC2.4 cells. I) IFN‐β production of 4T1 cells treated with different concentrations of EmDEX@GA and J) different groups of exosomes measured by enzyme‐linked immunosorbent assay (ELISA) (*n* = 3, **p < 0.01, ***p < 0.001). K) IFN‐β production of DC2.4 cells treated with different concentrations of EmDEX@GA and L) different groups of exosomes measured by ELISA (*n* = 3, **p < 0.01, ***p < 0.001).

The functions of EmDEX@GA were preliminarily tested in vitro. First, we co‐incubated EmDEX@GA with murine breast cancer cell line (4T1 cells) to test the PD‐1/PD‐L1 interactions between them. The results of flow cytometry showed that PD‐L1 detected on 4T1 cells gradually decreased with the increase of the concentration of EmDEX@GA (Figure S5, Supporting Information). While co‐incubated 4T1 cells with different groups of exosomes, apparent decrease of PD‐L1 detection was seen in both EmDEX and EmDEX@GA groups but not in DEX group (**Figure** **3**A,B). These results indicated the over‐expressed PD‐1 on EmDEX and EmDEX@GA could block the PD‐L1 on 4T1 cells thus decreasing the detection of it. In TDLNs, tumor‐derived substances would induce the occurrence of immunosuppressive microenvironment including high expression of PD‐L1 on DCs. To simulate the situation, we co‐incubated DC2.4 cells with the culture medium of 4T1 cells for 24 h and then treated them with different groups of exosomes. The results of flow cytometry demonstrated the expression of PD‐L1 on DC2.4 cells co‐incubated with the culture medium of 4T1 cells increased nearly twofold compared with untreated DC2.4 cells (Figure 3C,D). Moreover, the detections of PD‐L1 were greatly reduced in EmDEX and EmDEX@GA groups. Together, the capacity of PD‐1/PD‐L1 blockade of EmDEX@GA was proved in vitro.

Theoretically, the cellular uptake of EmDEX@GA could be conducted through typical endocytosis or the internalization triggered by PD‐1/PD‐L1 recognition.^[^ ^29^, ^36^ ^]^ By each way could the cGAMP loaded in exosomes get into cytoplasm to activate STING pathway. Figure 3E–H shows the cellular uptake of exosomes by 4T1 cells and DC2.4 cells. After co‐incubation for 24 h, the uptake percentages of exosomes were about 10% in 4T1 cells and up to about 38% in DC2.4 cells, indicated that EmDEX@GA could be internalized and release the loaded cGAMP to cytoplasm. Serve as the specific upstream ligand of STING, cGAMP could directly bind to and activate the STING pathway. Type I interferons including IFN‐β were the direct product of STING pathway that could reflect the activation of the pathway. So next, we tested the level of IFN‐β produced by cells treated with different groups of exosomes. Figure 3I‐L shows the production of IFN‐β was promoted by EmDEX@GA and displayed concentration‐dependent increase in 4T1 and DC2.4 cells. Compared with DEX and EmDEX, the cGAMP loaded in EmDEX@GA upregulated the level of IFN‐β for nearly twofold in both 4T1 and DC2.4 cells, which also indicated EmDEX@GA were effective for STING activation in vitro. Additionally, we tested the cytotoxicity of EmDEX@GA and found the cell viability had no obvious difference in exosomes treated groups compared with control group (Figures S6 and S7, Supporting Information). Which means the functions of EmDEX@GA were exerted by immunomodulation only, which would do no harm to immunocytes but activating them.

### 2.3 Active Targeting and Accumulation of EmDEX@GA in TDLNs

**Figure 4:**
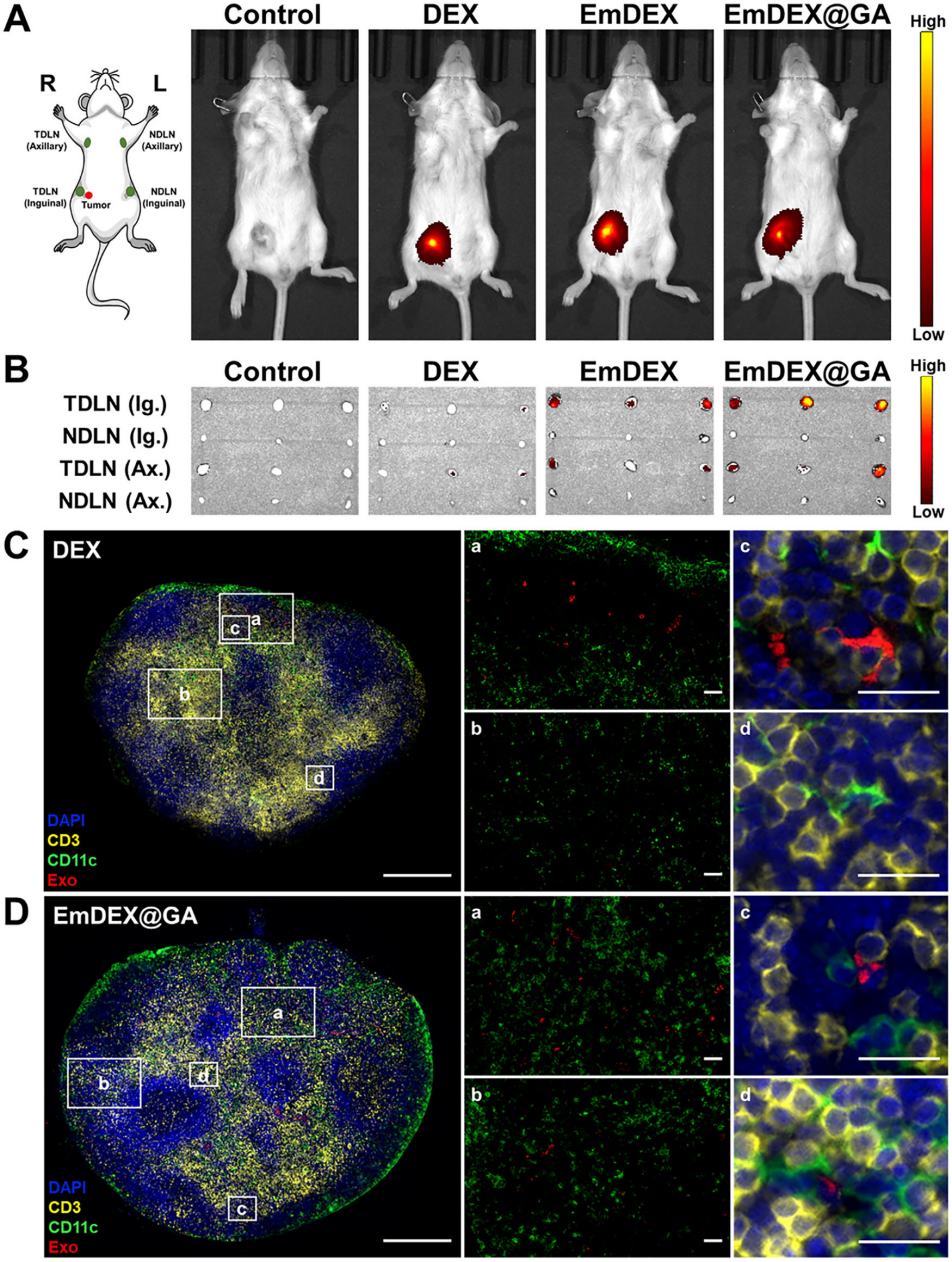
LN homing capacity of EmDEX@GA. A) Representative in vivo fluorescent images of mice intratumorally injected different groups of exosomes. B) Ex vivo fluorescent images of bilateral inguinal (Ig.) and axillary (Ax.) LNs of mice. C,D) Representative immunofluorescence images of inguinal TDLN of mice in C) DEX group and D) EmDEX@GA group (blue: DAPI; yellow: CD3; green: CD11c; red: DiR‐labeled exosomes; scale bar: 500 µm, 50 µm for (a, b), 20 µm for (c, d).

After characterizing the PD‐1/PD‐L1 blockade and STING pathway activation capacities of EmDEX@GA, we verified their performance on CCR7‐dependent active targeting to TDLNs. First, we established orthotopic breast tumor at the right fourth mammary gland of mice. The right inguinal LN and its downstream axillary LN were the TDLNs anatomically. Comparatively, the left inguinal and axillary LNs were set as NDLNs of the tumor. When the tumor grew to nearly 100 mm^3^, we labeled different groups of exosomes with fluorescent dye 1,1'‐dioctadecyl‐3,3,3',3'‐tetramethylindotricarbocyaine iodide (DiR) and intratumorally administrated these exosomes. In vivo fluorescent imaging at 24 h post‐injection showed adequate accumulation of exosomes in tumor (**Figure** **4**A). Then we took out the bilateral inguinal and axillary LNs of mice for exploring the accurate location of exosomes. Ex vivo imaging directly exhibited the larger size of TDLNs compared with their corresponding NDLNs (Figure 4B), which was consistent with the theoretical changes of TDLNs previously mentioned. Compared with DEX group, there were obvious accumulations of EmDEX and EmDEX@GA in TDLNs, represented their excellent LN homing capacity owing to the highly expressed CCR7. Immunofluorescence analysis was performed to further explore the effects of CCR7 and PD‐1 on the distribution of exosomes inside TDLN (Figure 4C,D; Figures S8 and S9, Supporting Information). Although DEX could get into TDLN through the lymphatic draining more or less, they had poor dispersivity and remained in cortex (Figure 4C). However, EmDEX and EmDEX@GA migrated to the paracortex of TDLN (Figure 4D; Figure S9, Supporting Information), where located DCs and T cell zones, indicated their possibility of PD‐1/PD‐L1 blockade between DCs and T cells. All of these results verified that EmDEX@GA had excellent TDLN targeting and great potential for local TDLN drug delivery.

### 2.4 Immunosuppressive Microenvironment of TDLN was Remodeled by EmDEX@GA

**Figure 5:**
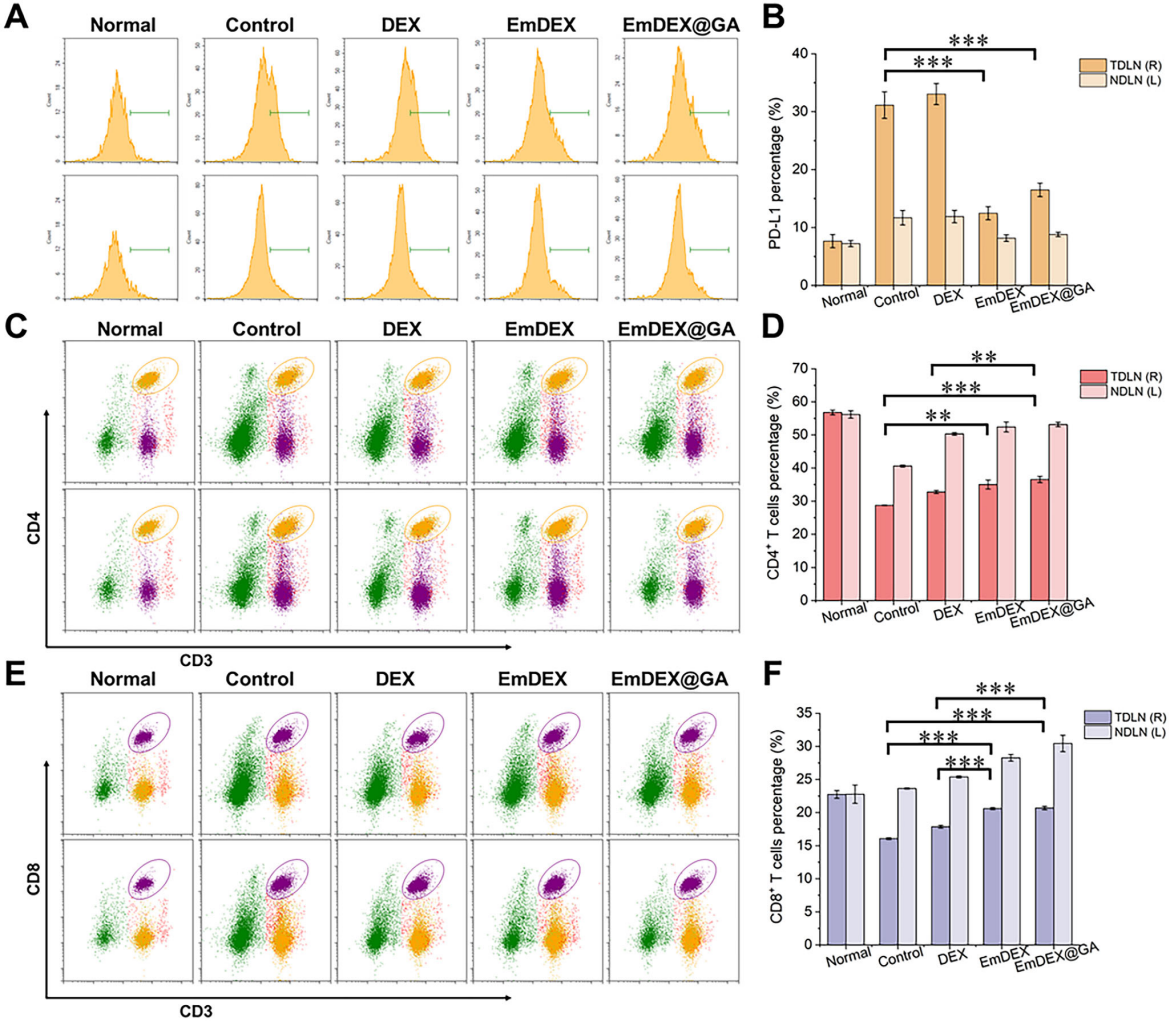
Remodeling of the immune microenvironment of TDLN. A) PD‐L1 expression on mature DCs in TDLN (right Ig.) and NDLN (left Ig.) and B) quantitative analysis by flow cytometry (*n* = 3, ***p < 0.001). C–F) Representative flow cytometric images and quantitative analysis of the percentages of C,D) CD4^+^ T cells and E,F) CD8^+^ T cells in TDLN (right Ig.) and NDLN (left Ig.) (*n* = 3, **p < 0.01, ***p < 0.001).

Since EmDEX@GA could migrate to and accumulate in TDLN, we analyzed their effects on modulating the immune microenvironment of TDLN. Bilateral inguinal LNs of breast tumor‐bearing mice were collected 24 h after intratumoral administration. Flow cytometry was conducted and constructive results were found. First, compared with healthy normal mice without tumor, the percentages of mature DCs were both increased in bilateral inguinal LNs of tumor‐bearing mice (Figure S10, Supporting Information). Especially, the percentage of mature DCs in TDLN was higher than NDLN, indicated the basic anti‐tumor efforts occurred in TDLN. However, the PD‐L1 expression on mature DCs in TDLN was nearly three times higher than NDLN and four times higher than normal LN (**Figure** **5**A,B), which might be associated with the dysfunction on antigen‐presenting of DCs. On the contrary, the percentages of CD4^+^ T‐helper cells and cytotoxic CD8^+^ T lymphocytes in TDLN were both sharply decreased compared with normal LN, for about 28% and 6%, respectively (Figure 5C–F). This connection between the numbers of T cells and the PD‐L1 expression on DCs verified the previous point that DC‐derived PD‐L1 would restrict the proliferation of T cells. On this condition, administration of different groups of exosomes showed distinct effects on the immune microenvironment of LNs. Due to the lacking of over‐expressed CCR7 and PD‐1, DEX made no difference on the specific blockade of PD‐L1 in LNs (Figure 5A,B). In contrast, EmDEX and EmDEX@GA demonstrated distinguished PD‐1/PD‐L1 blockade in TDLN for decreasing the PD‐L1 detection from more than 30% to about 15%. Even the PD‐L1 detections in NDLN were slightly decreased in these two groups that might correlated with the further lymphatic draining of exosomes driven by CCR7‐dependent LN targeting. Besides, owing to the load of cGAMP, percentage of mature DC in EmDEX@GA treated group was elevated from 30% to nearly 46% in TDLN (Figure S10, Supporting Information). The level of IFN‐β was correspondingly elevated, which indicated the STING pathway was activated by cGAMP (Figure S11A, Supporting Information). At the aspect of mechanisms, we performed investigation on the expression alterations associated with the STING signaling pathway. Figure S12A (Supporting Information) shows the expressions of phosphorylated STING and its downstream TANK‐binding kinase 1 (TBK1) and interferon regulatory factor 3 (IRF3) were upregulated in TDLN by EmDEX@GA, confirmed the successful activation of STING pathway in immunocytes including DCs. As for T cells, the numbers of CD4^+^ T cells and CD8^+^ T cells increased in DEX group, which could be attributed to the exogenous antigen carried on DEX that activated the basic immunity to a certain extent (Figure 5C–F). Combining this basic immunity with the relief of PD‐1/PD‐L1 restriction, the numbers of CD4^+^ T cells and CD8^+^ T cells in TDLN were higher elevated in EmDEX and EmDEX@GA groups. Especially for CD8^+^ T cells, the percentage of which was elevated from 16% to 20%, close to its normal value in healthy LN. The two types of T cells in NDLN appeared similar trends of increase, indicated the activation of TDLN enhanced systemic anti‐tumor immunity and proved the important role of TDLN in eliciting sufficient anti‐tumor immune responses. By the way, percentage of Tregs did not show obvious distinction in all the groups with treatments, suggested the exosomes had no influence on Tregs (Figure S13, Supporting Information). After analyzing the numbers of T cells, we then measured the IFN‐γ secretion in LNs to figure out whether the functions of T cells were activated. As shown in Figure S11B (Supporting Information), the levels of IFN‐γ in LNs of EmDEX and EmDEX@GA groups were visibly higher than other groups. It is typically that cytotoxic CD8^+^ T cells could produce large amounts of IFN‐γ directly involved in killing tumor cells.^[^ ^37^ ^]^ The higher level of IFN‐γ represented the more powerful anti‐tumor immunity. Furthermore, similarly serve as markers of the activation of T cells, the levels of tumor necrosis factor α (TNF‐α) and interleukin 12 (IL‐12) were also elevated by EmDEX@GA (Figure S12B,C, Supporting Information). These results supported the assume that local blockade of PD‐1/PD‐L1 interactions in TDLN could promote the activation and proliferation of T cells, thus eliciting potent anti‐tumor immunity. In summary, the remodeling of immune microenvironment in TDLN indicated the availability of EmDEX@GA for further suppressing tumor progression and metastasis.

### 2.5 EmDEX@GA Suppressed Tumor Progression by Enhancing Anti‐Tumor Immunity

For the EmDEX@GA were designed for local intratumoral administration, it could accumulate in both tumor and TDLN as the results exhibited above. In addition to verifying the effects of EmDEX@GA in TDLN, exploring its functions in tumor is equally important. We measured the levels of IFN‐β and IFN‐γ in tumor tissue after intratumoral administration for 24 h and found both of them increased in EmDEX@GA group (Figure S14A–C, Supporting Information). The increase of these cytokines indicated the activation of STING pathway and the potent functions of cytotoxic CD8^+^ T cells. Besides, the percentage of PD‐L1 in tumor was detected. After treatment with EmDEX@GA, the percentage of PD‐L1 decreased from 67% to 34%, demonstrated that PD‐1/PD‐L1 blockade happened in TME (Figure S14D,E, Supporting Information). Subsequently, the appropriate dosing regimen of EmDEX@GA was explored. In consideration of the characteristics of organismic immune responses, we performed low‐dose, high‐dose and low‐dose cycle administrations on orthotopic breast cancer mouse model, respectively (Figure S15A, Supporting Information). Different doses of EmDEX@GA were administrated at day 6. For low‐dose cycle group, the second and third administrations were given every 3 days. Tumor tissues were collected at day 7 and day 14 to analyze the levels of immune‐related cytokines. As shown in Figure S15 (Supporting Information), the productions of IFN‐β and IFN‐γ were elevated by EmDEX@GA under the effects of PD‐1/PD‐L1 blockade and STING pathway activation at day 7 in all treated groups, especially in the high‐dose group. However, the levels of IFN‐β and IFN‐γ in low‐dose cycle group became the highest at day 14. The productions of TNF‐α and IL‐12 in low‐dose cycle group were also the highest. These results implied that although the cytokines were temporarily elevated especially in high‐dose group after administration, the low‐dose cycle administration had the optimal effects on activating the sustained immune responses, which was corresponding to the phased immunotherapy regimen in clinical practice. Therefore, the low‐dose cycle administration regimen was chosen for further treatment.

**Figure 6:**
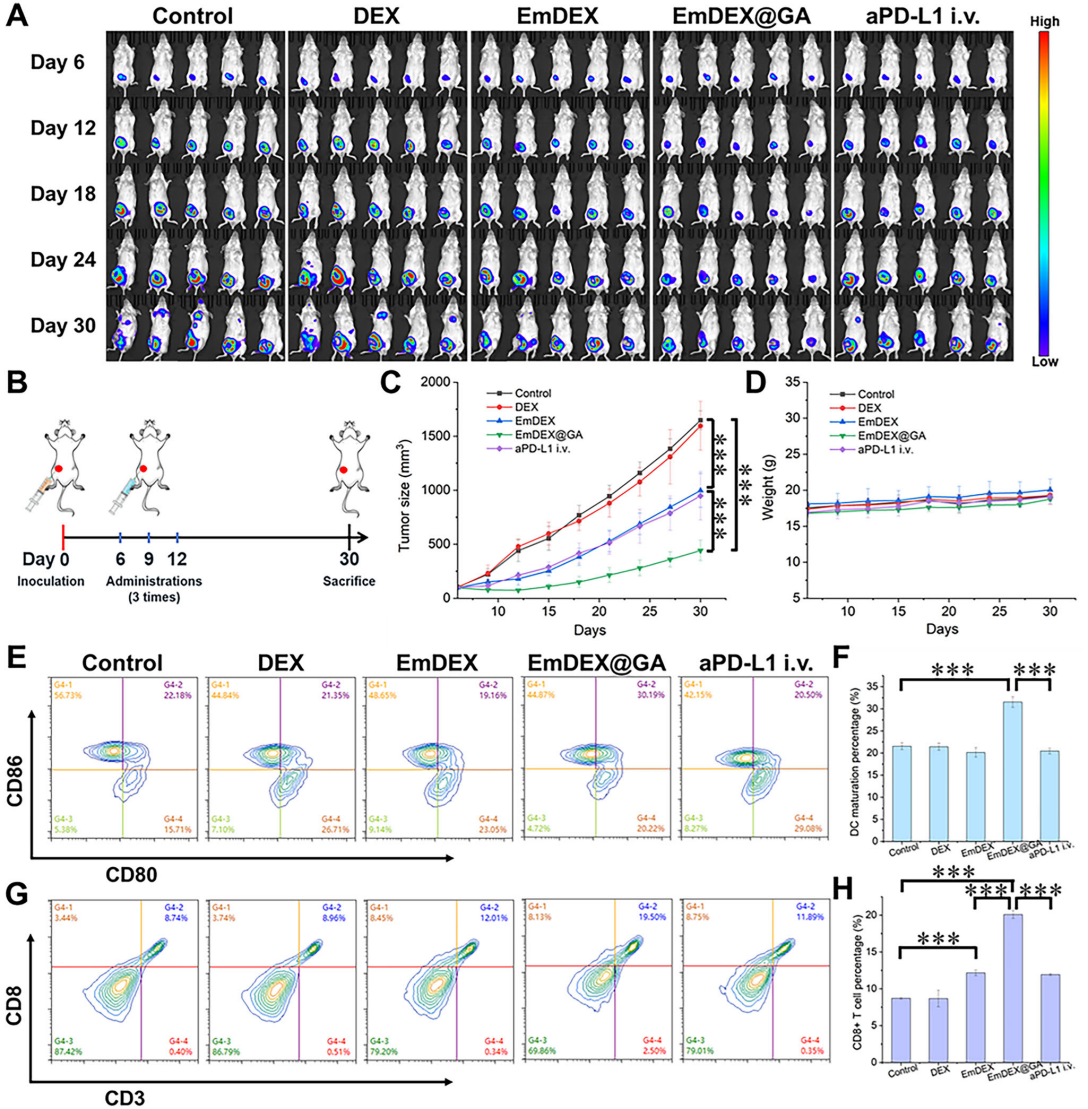
Local administration of EmDEX@GA for suppressing tumor progression. A) In vivo bioluminescent images of mice bearing orthotopic 4T1 tumor at set timepoints with different treatments. B) Schematic diagram of the timeline in mice administration study. C) Average tumor growth curves of 4T1 tumor‐bearing mice received different treatments (*n* = 5, ***p < 0.001). D) Average weight curves of mice in different groups. E–H) Representative flow cytometric images and quantitative analysis of the percentages of E, F) mature DCs and G, H) cytotoxic CD8^+^ T cells in tumor from different groups of mice (*n* = 3, ***p < 0.001).

Following a series of validations, EmDEX@GA were applied on orthotopic breast cancer mouse model to test their actual effects on suppressing tumor progression and metastasis. Except the four groups of control, DEX, EmDEX and EmDEX@GA intratumoral (i.t.) administration mentioned above, we added the fifth group, intravenous injection of anti‐PD‐L1 antibodies (aPD‐L1 i.v.), to further compare the therapeutic efficacy of locoregional ICB with systemic ICB. The mouse model was established by inoculating 4T1‐Luc tumor cells to the right fourth mammary gland of mouse. The first administration was given at 6 days after tumor implantation, by the time the volume of tumor had grown to around 100 mm^3^. The second and last administrations were given at day 9 and day 12 to boost the efficacy of immunotherapy. The situation of tumor progression and metastasis monitored by in vivo bioluminescent imaging was presented in **Figure** **6**A. Till day 30, tumor progression occurred in all groups but there was extremely severe metastasis in control and DEX groups. Besides, slight metastasis was seen in EmDEX and aPD‐L1 i.v. groups. Mice treated with EmDEX@GA did not show metastasis in the whole‐body bioluminescent imaging, which suggested the superiority of local administration of EmDEX@GA. Variation of tumor size measured at set timepoints demonstrated the growth rates of tumor decelerated in EmDEX, EmDEX@GA and aPD‐L1 i.v. groups (Figure 6C; Figure S16, Supporting Information). Especially, EmDEX@GA group had the lowest growth rate and minimal tumor size below 500 mm^3^ at day 30. In contrast, tumor size of control group grew to beyond 1500 mm^3^ at day 30, nearly threefold compared with EmDEX@GA group. The tumor growth inhibitions of EmDEX, EmDEX@GA and aPD‐L1 i.v. groups were 39.4%, 73.2% and 42.5%, respectively. These results indicated that local administration of EmDEX@GA had the optimum effects on suppressing tumor progression. In the meanwhile, the weight variation of mice had almost no difference among all groups (Figure 6D). We next analyzed the status of immunity inside tumor. The percentage of mature DCs and level of IFN‐β in EmDEX@GA group were remarkably elevated, which means EmDEX@GA could activate STING pathway to produce type I interferons and promote DC maturation (Figure 6E,F; Figure S17A, Supporting Information). Simultaneously, percentages of cytotoxic CD8^+^ T cells were increased in EmDEX, EmDEX@GA and aPD‐L1 i.v. groups (Figure 6G,H). Among these three groups, tumor received EmDEX@GA treatment had the most amounts of CD8^+^ T cells, which was twofold compared with control group. The production of IFN‐γ in tumor showed corresponding alterations in different groups (Figure S17B, Supporting Information). Together, the results suggested EmDEX@GA enhanced anti‐tumor immunity owing to the combination of PD‐1/PD‐L1 blockade and STING pathway activation, which consequently suppressed tumor progression.

It is worth noting that even if the anti‐tumor efficacy of EmDEX i.t. and aPD‐L1 i.v. seemed similarly, they still had underlying distinctions. To suit the characteristics of systemic administration, the dosage of aPD‐L1 was at least two times higher than EmDEX applied on each mouse in corresponding groups. These results suggested locoregional PD‐1/PD‐L1 blockade in TME and TDLN achieved similar anti‐tumor efficacy with lower dosage and fewer side effects compared with systemic ICB. Given that the poor enrichment in LNs of systemic administration, the essential role of TDLNs in suppressing tumor progression was proved.

### 2.6 EmDEX@GA Reduced the Incidences of LNM and DM

**Figure 7:**
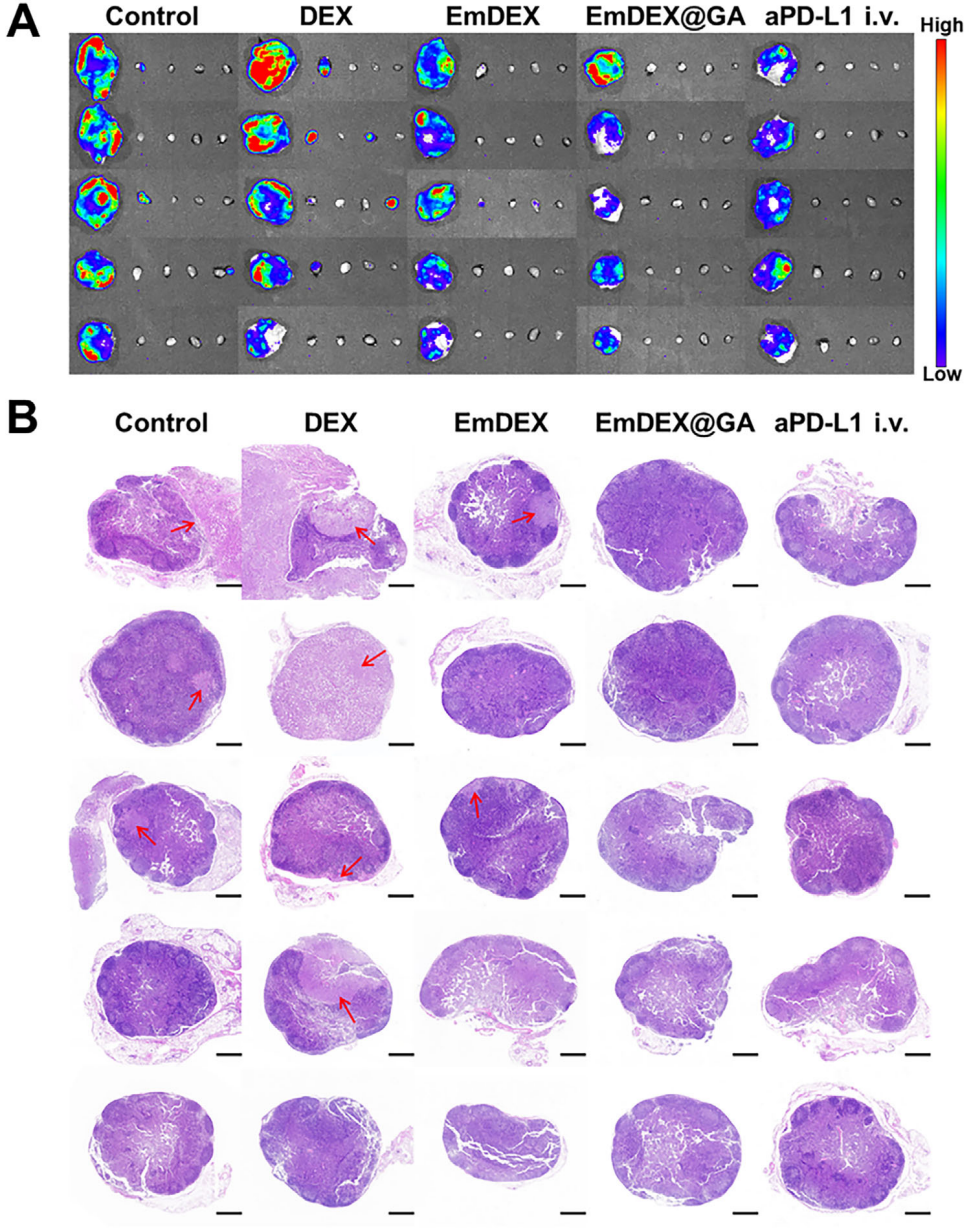
Suppression of LNM by EmDEX@GA. A) Ex vivo bioluminescent images of tumor and bilateral inguinal and axillary LNs harvested from mice in different groups. B) H&E staining images of inguinal TDLN of mice in different groups. The location of metastasis was pointed by red arrow (scale bar: 500 µm).

To deeply explore the effects of EmDEX@GA on suppressing tumor metastasis, we harvested the tumor, bilateral inguinal and axillary LNs, and major organs of each mouse in different groups for further analysis. Ex vivo bioluminescent imaging was performed to confirm the accurate location of LNM (**Figure** **7**A). Additionally, LNs and organs were conducted H&E staining for avoiding the negative detection of micrometastasis in bioluminescent imaging (Figure 7B; Figure S18, Supporting Information). The incidence rates of LNM and DM were calculated by combining the results of imaging and pathology (**Table** **1**). The types of LNM were further divided into TDLN side (ipsilateral of tumor) and NDLN side (contralateral of tumor). DM was defined as at least one of the organs involved. According to the results, the incidences of LNM and DM were both high in control and DEX groups, with 80% LNM and 100% DM. The status of metastasis in these two groups was so severe that represented quite a late stage of tumor. However, in EmDEX group, the incidence of LNM decreased to 40% and all occurred in TDLN side. The incidence of DM even decreased to 20%, indicated great amelioration of tumor metastasis. With the combination of PD‐1/PD‐L1 blockade and STING pathway activation in TME and TDLN, mice in EmDEX@GA group had no detectable metastasis in LNs or organs, demonstrated the significant work of EmDEX@GA on suppressing tumor metastasis. Interestingly, we found the incidence of LNM decreased to 20% in aPD‐L1 i.v. group, but the incidence of DM was 60%. Distant metastasis is commonly seen as more severe index than LNM in clinical TNM tumor stage. The occurrence of DM mostly represents the tumor has progressed to stage IV with extremely poor prognosis. Therefore, although the effects on suppressing tumor growth of EmDEX i.t. and aPD‐L1 i.v. seemed similarly as previously mentioned, the high incidence of DM in aPD‐L1 i.v. group suggested that local administration of EmDEX achieved better efficacy on suppressing tumor metastasis. These results highlighted the excellent potential of EmDEX@GA in reducing the incidences of LNM and DM, and once again proved that locally remodeled the immune microenvironment of TDLNs made a great difference in suppressing tumor progression and metastasis.

**Table 1:** The incidence rates of LNM and DM.

	LNM	LNM (TD)	LNM (ND)	DM
Control	80%	60%	40%	100%
DEX	80%	80%	20%	100%
EmDEX	40%	40%	0	20%
EmDEX@GA	0	0	0	0
aPD‐L1 i.v.	20%	20%	0	60%

### 2.7 The TDLN Immune Microenvironment Plays an Essential Role in the Occurrences of Distant Metastasis

**Figure 8:**
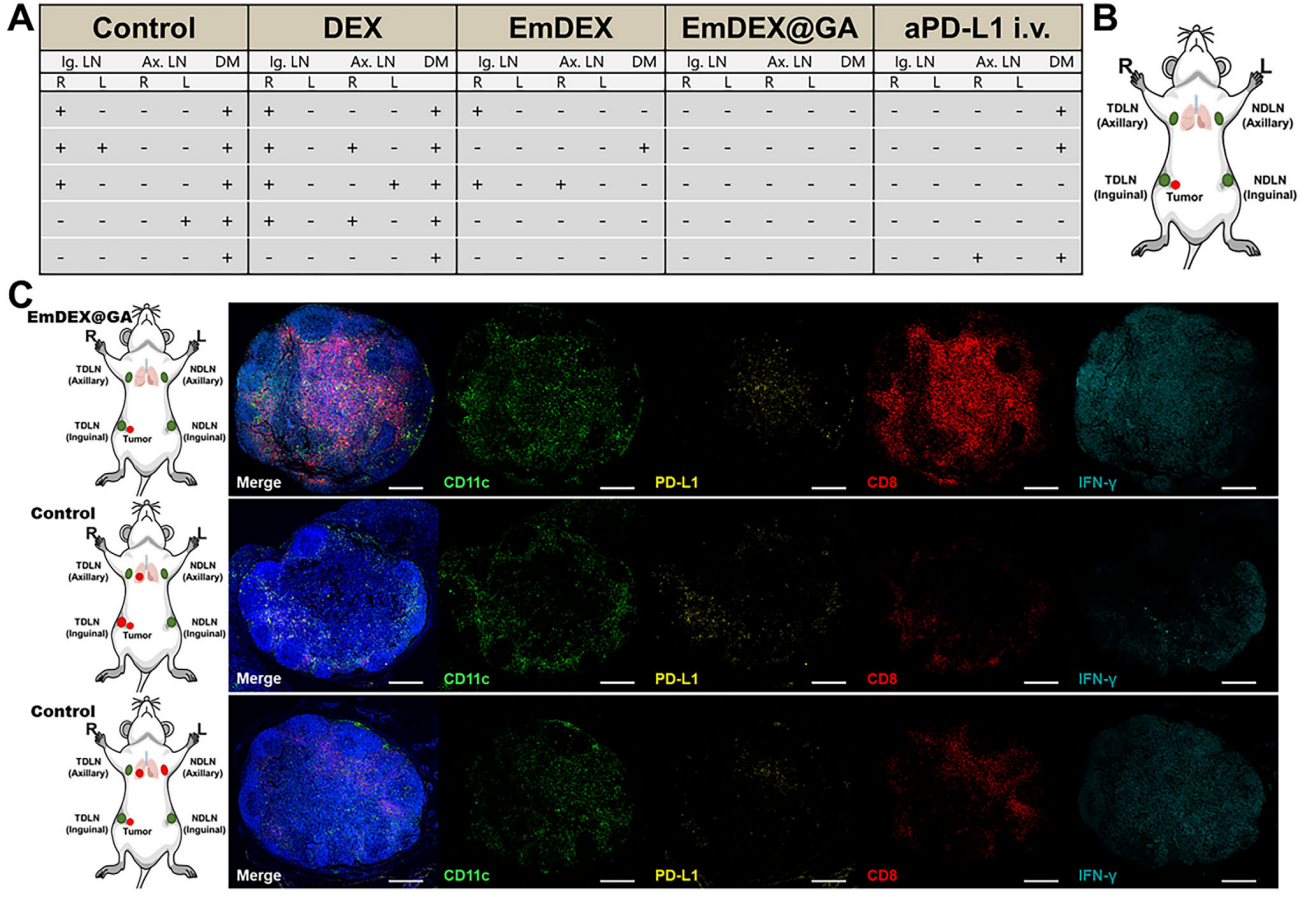
The role of TDLN immune microenvironment in tumor metastasis. A) Summative diagram of LNM and DM status of mice in different groups (+: positive; ‐: negative). B) Schematic diagram of the LN sites in mice. C) Schematic diagram of the metastatic status of mouse (red: tumor or metastasis; green: negative) and the corresponding immunofluorescence images of the inguinal TDLN (blue: DAPI; green: CD11c; yellow: PD‐L1; red: CD8; cyan: IFN‐γ; scale bar: 500 µm).

While analyzing the accurate location of metastasis, we found something interesting that worth further explored. The detailed metastatic status of each mouse in different groups was schematized in **Figure** **8**A,B. We could tell that the order of occurrence within LNM and DM was quite complicated from these data. In the orthotopic breast cancer mouse model we established, tumor was inoculated to the right fourth mammary gland. Thus, the right inguinal LN was the nearest of the TDLNs anatomically, and then the right axillary LN lay on the downstream site.^[^ ^38^ ^]^ The left inguinal and axillary LNs were far from the draining of tumor. The results in Figure 8A show that some of the LNM only occurred in the nearest TDLN, some occurred in both right inguinal and axillary LNs in tumor‐draining side. However, there were conditions that metastasis occurred in distant NDLNs without TDLN involved, which made the behavior of LNM became mysterious. It seemed that tumor metastasis in LNs did not appear a linear, sequential mode as we conventionally understood. In addition, distant metastasis in organs could occur without LNM (Figure 8A), which kind of supported the perspective that visceral metastasis could occur coincident with or without LNM.^[^ ^39^ ^]^ In consideration of the significance of TDLN immune microenvironment in systemic anti‐tumor immunity demonstrated above. We analyzed the immune microenvironment of the certain TDLN associated with these particular behaviors of metastasis. Immunofluorescence images of inguinal TDLN and the status of metastasis in the certain mouse are shown in Figure 8C. After a series of local EmDEX@GA administration, the amounts of DCs, cytotoxic CD8^+^ T cells and the production of IFN‐γ in the right inguinal LN (the closest TDLN of tumor) were obviously higher than that in control group. Although PD‐L1 still expressed on DCs to some extent, these results represented the anti‐tumor immunity elicited by early treatments of EmDEX@GA was persistently effective, which contributed to the later suppression of LNM and DM. As for the mouse in control group that had metastasis in organs and inguinal TDLN, the microenvironment in TDLN was extremely immunosuppressive (Figure 8C). The immunosuppressive microenvironment in TDLN means weak export of immunocytes and cytokines, in company with the impaired systemic anti‐tumor immunity, subsequently resulting in the occurrence of distant metastasis. However, in the mouse only had distant metastasis in NDLNs and organs, we surprisingly found the similar immunosuppressive microenvironment in its inguinal TDLN, even if there was not regional metastasis inside it. It was such kind of alteration that contributed to the non‐sequential occurrences of LNM and DM. The immunosuppressive microenvironment of TDLNs had already weakened the systemic anti‐tumor immunity before the formation of regional metastasis in TDLNs. Therefore, distant metastasis could occur simultaneously or earlier than the metastasis in TDLNs. It was the immune microenvironment rather than the metastatic status in TDLNs that associated with distant metastasis. Thus, for the mice whose immunosuppressive microenvironment of TDLN was remodeled by EmDEX@GA, the incidence of distant metastasis was reduced as the results shown in Table 1. This inference highlighted the particularity of TDLN for concurrently as an immune organ and the site of metastasis. All above, the immune microenvironment of TDLNs is essential to the occurrences of distant metastasis, remodeling the immunosuppressive microenvironment of TDLNs is meaningful for suppressing tumor progression and metastasis.

## 3 Conclusion

With the gradually intensive research of tumor immunotherapy, studies have found that TDLNs were the crossroad of tumor immunity and evasion. The immune checkpoint interactions in TDLNs correlated with the priming of anti‐tumor immunity. In this study, we verified increased expression of immunosuppressive ligand PD‐L1 on DCs and the correspondingly weak anti‐tumor immunity in TDLNs. The engineered exosomes EmDEX@GA we constructed could actively target to TDLNs via intratumoral administration, remodel the immunosuppressive microenvironment of TDLNs by PD‐1/PD‐L1 blockade and STING‐mediated immune activation. These actions resulted in the enhancement of systemic anti‐tumor immunity, which subsequently suppressed tumor progression as well as the incidences of LNM and DM. Especially compared with systemic anti‐PD‐L1 immunotherapy, the locoregional PD‐1/PD‐L1 blockade and immunomodulation of TDLN by EmDEX@GA exhibited more effective suppression on LNM and DM, which indicated the essential role of TDLNs in tumor progression and metastasis apart from TME. Moreover, we found the immune microenvironment in TDLNs, but not the metastatic status of them, was closely associated with the occurrences of distant metastasis. This association further confirmed the rationality of targeting TDLNs for suppressing metastasis and improving prognosis. Although the deeper mechanisms between the immunosuppressive TDLNs and metastasis still need to be explored, the results of our study proved the strategy of local TDLN immunomodulation by EmDEX@GA to suppress tumor progression and metastasis is feasible. It is promising for clinical translation and further combination with minimally invasive therapy or targeted immunotherapy to reinforce the efficacy of tumor comprehensive therapy.

## 4 Experimental Section

### Materials

Dulbecco′s Modified Eagle Medium (DMEM) and fetal bovine serum (FBS) were purchased from Thermo Fisher Scientific (Waltham, USA). Penicillin‐streptomycin was purchased from HyClone (Logan, USA). Puromycin, BCA protein assay kit and skimmed milk powder were obtained from Beyotime Biotechnology (Shanghai, China). Lipopolysaccharide (LPS) was obtained from Sigma–Aldrich (Missouri, USA). 2′,3′‐cGAMP (cGAMP) was purchased from InvivoGen (Hong Kong, China). 2′,3′‐cGAMP enzyme‐linked immunosorbent assay (ELISA) kit was obtained from Cayman Chemical (Ann Arbor, USA). 3,3'‐dioctadecyloxacarbocyanine perchlorate (DiO), 1,1'‐dioctadecyl‐3,3,3',3'‐tetramethylindotricarbocyaine iodide (DiR) and anti‐mouse PD‐L1 antibody were purchased from MedChemExpress (Shanghai, China). Mouse IFN‐β ELISA kit, mouse IFN‐γ ELISA kit, mouse TNF‐α ELISA kit and mouse IL‐12/P40 ELISA kit were purchased from CUSABIO (Wuhan, China). Cell counting kit‐8 (CCK‐8) was obtained from NCM Biotech (Suzhou, China). RIPA lysis buffer was obtained from Solarbio Life Science (Beijing, China). Sodium dodecyl sulfate‐polyacrylamide gel electrophoresis (SDS‐PAGE), protein loading buffer, protein marker and chemiluminescent kit were purchased from Epizyme Biotech (Shanghai, China). Polyvinylidene fluoride (PVDF) membrane and ultra filters were purchased from Millipore (Billerica, USA). Primary antibodies of western blot including anti‐PD‐1 antibody and anti‐CCR7 antibody were purchased from Abcam (Cambridge, UK), GAPDH antibody and STING/TMEM173 antibody were purchased from ABclonal Biotechnology (Wuhan, China), phospho‐TMEM173/STING antibody, phospho‐TBK1 antibody, phospho‐IRF3 antibody and β‐Actin antibody were purchased from Affinity Biosciences (Jiangsu, China), anti‐CD63 antibody was purchased from Solarbio Life Science (Beijing, China). Horseradish peroxidase (HRP)‐conjugated secondary antibody was obtained from Boster Biological Technology (Wuhan, China). Antibodies for flow cytometry including APC anti‐mouse PD‐1 antibody, FITC anti‐mouse PD‐1 antibody, PE/Cyanine7 anti‐mouse CCR7 antibody, FITC anti‐mouse CCR7 antibody, PerCP/Cyanine5.5 anti‐mouse PD‐L1 antibody, PE/Cyanine7 anti‐mouse PD‐L1 antibody, FITC anti‐mouse CD11c antibody, PE/Cyanine7 anti‐mouse I‐A/I‐E (MHCII) antibody, PerCP/Cyanine5.5 anti‐mouse CD80 antibody, APC anti‐mouse CD86 antibody, FITC anti‐mouse CD3 antibody, APC anti‐mouse CD4 antibody, PE/Cyanine7 anti‐mouse CD8a antibody, and PE anti‐mouse FOXP3 antibody were all purchased from BioLegend (San Diego, USA). D‐luciferin potassium was obtained from Macklin (Shanghai, China).

### Cell Culture and Mice

Murine breast cancer cell line (4T1 cells) and murine dendritic cell line (DC2.4 cells) were obtained from EK‐bioscience Biotechnology Co. Ltd (Shanghai, China). 4T1‐Luc cells were constructed by transfecting luciferase reporter gene into 4T1 cells followed by positive selection. Cells were cultured in DMEM supplemented with FBS (10%) and penicillin‐streptomycin (1%) at 37 °C in incubator containing CO_2_ (5%). For exosomes isolation, cells were cultured in DMEM supplemented with exosome‐depleted FBS (10%) and penicillin–streptomycin (1%).

Female BALB/c mice (5–6 weeks) were purchased from BesTest Biotechnology Co. Ltd (Zhuhai, China). All the animal experiments were approved by the Institutional Animal Care and Use Committee, Sun Yat‐Sen University (SYSU‐IACUC‐2024‐002597).

### Construction of Parental DCs

The parental DCs with PD‐1 and CCR7 over‐expression were constructed first. Lentivirus package of plasmid with gene encoding PD‐1 was conducted by VectorBuilder Inc. (Guangzhou, China). For lentivirus transfection, DC2.4 cells were co‐incubated with the constructed lentiviruses for 24 h and selected by puromycin. The positive transfected cells were maintained by puromycin and induced maturation by treating with LPS (5µg mL^−1^) for 24 h. The maturation percentages of DCs were analyzed by flow cytometry. PD‐1 and CCR7 over‐expressions were confirmed by flow cytometry and WB analysis.

### Preparation and Characterization of Exosomes

Exosomes DEX were isolated from the culture supernatant of DC2.4 cells, and EmDEX were isolated from the culture supernatant of DC2.4 cells after lentivirus transfection and LPS inducement (EmDC). Different kinds of cell culture supernatants were collected after culturing for 48 h and received sequential centrifugation. Briefly, supernatants were centrifuged at 300 and 2000 g for 10 min each, followed by 10 000 g for 30 min and ultracentrifugation at 100 000 g for 90 min to isolate exosomes. The exosomes pellets were washed by phosphate‐buffered saline (PBS) and received ultracentrifugation at 100 000 g for another 90 min. After that, exosomes were resuspended in PBS for further study. To obtain exosomes EmDEX@GA, EmDEX were incubated with cGAMP (1:1) with rotation for 16 h at room temperature and then washed with PBS by using 30 kDa ultra filter according to manufacturer′s instructions. The drug loading of cGAMP was measured by detecting the quantity of cGAMP in the lysis of EmDEX@GA by ELISA kit. The concentration of exosomes was measured by BCA protein assay kit followed instructions. Furthermore, exosomes were stained with uranyl acetate and observed by TEM (HITACHI, HT7800, Japan). The size of exosomes was measured by NTA instrument (Particle Metrix, ZetaView, Germany). After being preserved at 4 and 37 °C for 24 h, the size stability of exosomes was measured by Malvern Zeta‐sizer (Malvern, Nano ZS90, UK). Specific protein expression of exosomes was analyzed by WB. For the expression percentages of PD‐1 and CCR7, DEX and EmDEX@GA were marked by the corresponding fluorescence‐conjugated antibodies and analyzed by Flow NanoAnalyzer (NanoFCM, U30E, China).

### In Vitro Analysis of PD‐1/PD‐L1 Blockade

4T1 cells were seeded in 6‐well plates for attachment. Different concentrations of EmDEX@GA (0, 12.5, 25, 50, and 100 µg mL^−1^) as well as different groups of exosomes (50 µg mL^−1^; DEX, EmDEX, and EmDEX@GA) were added into and co‐incubated for 24 h. For control group, equal volume of PBS was added. Cells were harvested and analyzed by flow cytometry for the quantity of PD‐L1.

For the analysis of PD‐L1 expression and PD‐1/PD‐L1 blockade on DCs. Culture medium of 4T1 cells after culturing for 48 h was collected first and mixed with DMEM (with 10% FBS) at a ratio of 1:1. DC2.4 cells were seeded in 6‐well plates for attachment. Some of the wells were treated with the mixed medium above and incubated for 24 h. Then, these mixed medium treated cells were co‐incubated with different groups of exosomes (50 µg mL^−1^; DEX, EmDEX, and EmDEX@GA) for another 24 h. For control group, equal volume of PBS was added. Finally, DC2.4 cells untreated, or treated with 4T1 medium and exosomes were all harvested. The PD‐L1 expression on DCs was analyzed by flow cytometry.

### In Vitro Cellular Uptake of Exosomes

Exosomes were first incubated with DiO (0.5 mm) at a ratio of 100:1 in volume for 5 min at 37 °C. Then, the DiO‐labeled exosomes were washed with PBS by using 30 kDa ultra filter. DC2.4 and 4T1 cells were seeded in 6‐well plates, respectively. After attachment, cells were treated with DiO‐labeled DEX, EmDEX, and EmDEX@GA (50 µg mL^−1^). Equal volume of PBS was added in control group. After co‐incubation for 24 h, culture medium was discarded and cells were harvested for flow cytometry.

### In Vitro Evaluation of STING Pathway Activation

DC2.4 and 4T1 cells were seeded in 12‐well plates, respectively. After cell attachment, different concentrations of EmDEX@GA (0, 12.5, 25, 50, and 100 µg mL^−1^) as well as different groups of exosomes (50 µg mL^−1^; DEX, EmDEX, and EmDEX@GA) were added into and co‐incubated for 48 h. Equal volume of PBS was added in control group. The cell culture supernatants were collected and analyzed by IFN‐β ELISA kit.

### Cytotoxicity Evaluation

The cytotoxicity of exosomes was evaluated by CCK‐8 assay. DC2.4 and 4T1 cells were seeded in 96‐well plates, respectively. After attachment, cells were treated with different concentrations of EmDEX@GA (0, 12.5, 25, 50, and 100 µg mL^−1^) and different groups of exosomes (50 µg mL^−1^; DEX, EmDEX, and EmDEX@GA). For control group, equal volume of PBS was added. After co‐incubation for 24 h, CCK‐8 was added to cells and incubated for another 2 h. Cell viability was obtained by measuring the absorbance at 450 nm with a microplate reader (BioTek, Synergy H1, USA).

### Western Blot

Protein samples of cells and exosomes were extracted with RIPA lysis buffer, denatured and loaded into SDS‐PAGE (10%). Then, the segregated protein samples were transferred to a PVDF membrane followed by blocking in skimmed milk (5%) for 1 h. After that, the membrane was incubated with the corresponding primary antibodies overnight at 4 °C and further incubated with HRP‐conjugated secondary antibody for 2 h. The blots were observed by chemiluminescent imaging system (Bio‐Rad, ChemiDoc MP, China).

### Flow Cytometry

Single‐cell suspensions of cell samples were prepared and stained with corresponding fluorescence‐conjugated antibodies followed manufacturer′s instructions. The fluorescent signals of cell samples were detected by flow cytometer (Challenbio, FongCyte, China) and the data were analyzed with its accompanying software and FlowJo software.

### Accumulation of Exosomes in TDLNs

Exosomes were incubated with DiR (1 mm) at a ratio of 100:1 in volume for 5 min at 37 °C. Then, the DiR‐labeled exosomes were washed with PBS by using 30 kDa ultra filter. Orthotopic breast cancer mouse model was established. 4T1 tumor cells (1 × 10^6^) suspended in PBS (100 µL) were inoculated to the right fourth mammary fat pad of BALB/c mice. When the tumor grew to nearly 100 mm^3^, DiR‐labeled exosomes (50 µg) suspended in PBS (50 µL) were intratumorally administrated. Equal volume of PBS without exosomes was intratumorally administrated in control group. After 24 h of administration, mice were imaged by in vivo imaging system (IVIS; PerkinElmer, Lumina Series III, USA). Then, bilateral inguinal and axillary LNs of mice were resected and observed by IVIS system for the accurate location of exosomes. To confirm the distribution of DiR‐labeled exosomes inside TDLN, tissue sections of the right inguinal LNs were stained with several antibodies of cell markers (DAPI for nucleus; CD3 for T cells; CD11c for DCs) and performed immunofluorescence analysis.

### Immune Microenvironment Remodeling in TDLNs by Exosomes

Orthotopic breast cancer mouse model was established as previous description. When the tumor grew to nearly 100 mm^3^ (day 6), different groups of exosomes (50 µg; DEX, EmDEX, and EmDEX@GA) suspended in PBS (50 µL) were intratumorally administrated. PBS (50 µL) without exosomes was intratumorally administrated in control group. Healthy mice without tumor inoculation and any treatment were set as normal control group. After 24 h of administration, bilateral inguinal LNs of mice were resected and dissociated into single‐cell suspensions to perform flow cytometric analysis. Percentages of mature DCs (CD11c^+^ CD86^+^ MHCII^+^), PD‐L1 expression on mature DCs (CD11c^+^ MHCII^+^ PD‐L1^+^), CD4^+^ T cells (CD3^+^ CD4^+^ CD8^−^), CD8^+^ T cells (CD3^+^ CD8^+^ CD4^−^), and Tregs (CD3^+^ CD4^+^ FOXP3^+^) were calculated. For the validation of STING pathway activation, the relevant protein expression of cells in the inguinal TDLN of mice was analyzed by WB. Besides, to measure the levels of IFN‐β, IFN‐γ, TNF‐α and IL‐12 in LNs, bilateral inguinal LNs of mice were homogenized and analyzed by corresponding ELISA kit.

### Immune Responses Elicited by Exosomes in TME

Orthotopic breast cancer mouse model was established as previous description. When the tumor grew to nearly 100 mm^3^ (day 6), different groups of exosomes (50 µg; DEX, EmDEX, and EmDEX@GA) suspended in PBS (50 µL) were intratumorally administrated. PBS (50 µL) without exosomes was intratumorally administrated in control group. After 24 h of administration, tumor tissue samples were collected and digested to single‐cell suspensions. Percentage of PD‐L1 expression was analyzed by flow cytometry. To measure the levels of IFN‐β and IFN‐γ, tumor tissue samples in different groups were homogenized and analyzed by corresponding ELISA kit.

### Dosing Regimen Tests

Orthotopic breast cancer mouse model was established as previous description. The administrations were performed at day 6 (when the tumor grew to nearly 100 mm^3^). For low‐dose group, EmDEX@GA (50 µg) suspended in PBS (50 µL) were intratumorally administrated. For high‐dose group, EmDEX@GA (150 µg) suspended in PBS (50 µL) were intratumorally administrated. For low‐dose cycle group, EmDEX@GA (50 µg) suspended in PBS (50 µL) were intratumorally administrated at day 6, day 9, and day 12. For control group, PBS (50 µL) without exosomes was intratumorally administrated. Tumor tissue samples were collected at day 7 and day 14, respectively. To measure the levels of cytokines IFN‐β, IFN‐γ, TNF‐α and IL‐12, tumor tissue samples were homogenized and analyzed by corresponding ELISA kit.

### In Vivo Anti‐Tumor Therapy

Orthotopic breast cancer mouse model was established by inoculating 4T1‐Luc tumor cells (1 × 10^6^ suspended in 100 µL PBS) into the right fourth mammary fat pad of BALB/c mice. When the size of tumor approached nearly 100 mm^3^ (day 6), different treatments were given. DEX, EmDEX and EmDEX@GA exosomes (50 µg) suspended in PBS (50 µL) were intratumorally (i.t.) administrated to per mouse in corresponding groups. PBS (50 µL per mouse) without exosomes was i.t. administrated in control group. For aPD‐L1 i.v. group, anti‐PD‐L1 antibodies (1 µg µL^−1^; 100 µL) were intravenously (i.v.) injected via tail vein. The administrations were given every 3 days for total three times. Tumor size was calculated as length × width^2^ × 1/2, which was measured every 3 days along with the body weight of mice. The bioluminescence of orthotopic and metastatic tumor was monitored every 6 days. Briefly, D‐luciferin potassium (15 mg mL^−1^; 10 µL g^−1^) was intraperitoneally injected. After injection for 5 min, the bioluminescent imaging of mice was observed by IVIS system (PerkinElmer, Lumina Series III, USA).

### Anti‐Tumor Immunity and Metastatic Status Analysis

Mice in all groups were continuously monitored as described above and euthanized at day 30 (when the tumor size of control group exceeded 1500 mm^3^). The tumor, bilateral inguinal and axillary LNs, and major organs including heart, liver, spleen, lung, and kidney were collected for further analysis. First, tumor and LNs were performed ex vivo bioluminescent imaging by IVIS system (PerkinElmer, Lumina Series III, USA). Next, tumor tissue samples were digested to single‐cell suspensions and performed flow cytometric analysis. Percentages of mature DCs (CD11c^+^ CD80^+^ CD86^+^) and CD8^+^ T cells (CD3^+^ CD8^+^) were calculated. To measure the levels of IFN‐β and IFN‐γ, tumor tissue samples in different groups were homogenized and analyzed by corresponding ELISA kit. For the analysis of metastatic status, LNs and organs were embedded in paraffin and conducted H&E staining. Finally, to analyze the immune microenvironment of TDLN, the right inguinal LN tissue sample of the certain mouse was stained with antibodies of different markers (DAPI, CD11c, PD‐L1, CD8 and IFN‐γ) and performed immunofluorescence analysis.

### Statistical Analysis

Quantitative data were presented as means ± standard deviation (SD) of at least three samples and analyzed by one‐way analysis of variance (ANOVA) and Student′s *t*‐test. Values of *p < 0.05, **p < 0.01, and ***p < 0.001 were considered as statistically significant. The statistical analysis was conducted with SPSS Statistics software.

## Conflict of Interest

The authors declare no conflict of interest.

## Author Contributions

Y.W., X.G., and J.Q. contributed equally to this work. Y.W., X.G., and J.Q. conducted the main experiments and wrote the manuscript. Y.X., P.Z., Y.L., M.C., G.Z., and X.S. assisted with partial experiments. L.C. assisted with data analysis. B.L., J.L., and J.R. designed the experiments and supervised the writing.

## Supporting information



Supporting Information

## Data Availability

The data that support the findings of this study are available from the corresponding author upon reasonable request.
