# Supplementary material for: Locoregional Immune Checkpoint Blockade and Remodeling of Lymph Nodes by Engineered Dendritic Cell‐Derived Exosomes for Suppressing Tumor Progression and Metastasis
Source: Adv Sci (Weinh). 2025 Apr 3;12(23):2500139. doi: 10.1002/advs.202500139 (PMC12199396; doi:10.1002/advs.202500139)
Supplement: Supplementary file 1 — Supporting Information [file ADVS-12-2500139-s001.pdf]

ADVANCED SCIENCE
Open Access

Supporting Information

for *Adv. Sci.*, DOI 10.1002/advs.202500139

Locoregional Immune Checkpoint Blockade and Remodeling of Lymph Nodes by Engineered Dendritic Cell-Derived Exosomes for Suppressing Tumor Progression and Metastasis

Yizhen Wang, Xiaomin Guo, Jingya Qin, Yifan Xue, Peng Zhang, Yadong Liu, Moyang Chen, Guanghao Zhu, Xinqiu Song, Lili Cheng, Bo Liu, Jie Liu* and Jie Ren**

Supporting Information

Locoregional Immune Checkpoint Blockade and Remodeling of Lymph Nodes by Engineered Dendritic Cell-Derived Exosomes for Suppressing Tumor Progression and Metastasis

Yizhen Wang, Xiaomin Guo, Jingya Qin, Yifan Xue, Peng Zhang, Yadong Liu, Moyang Chen, Guanghao Zhu, Xinqiu Song, Lili Cheng, Bo Liu,* Jie Liu,* and Jie Ren*

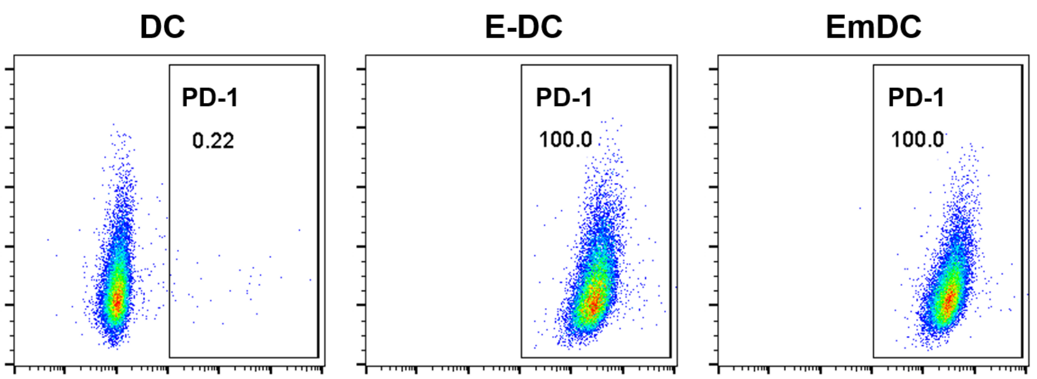

Figure S1. Flow cytometry analysis of PD-1 expression percentages of DC, E-DC and EmDC.

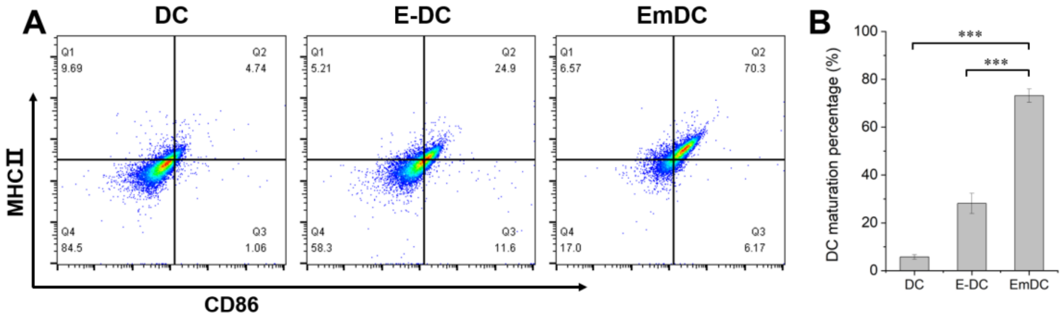

Figure S2. (A.B) Flow cytometry analysis of the maturation percentages of DC, E-DC and EmDC ($n = 3$, ***$P < 0.001$).

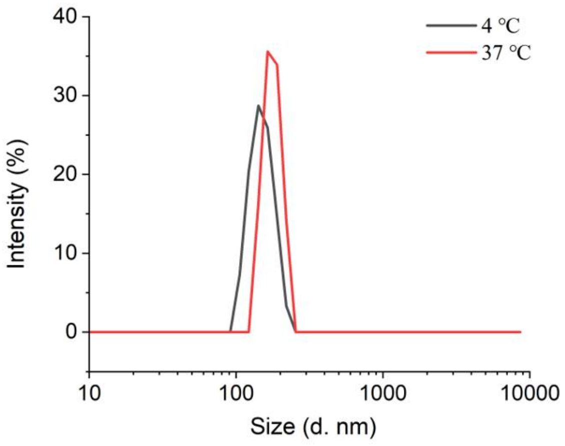

Figure S3. Size distribution of EmDEX@GA at 4 ℃ and 37 ℃.

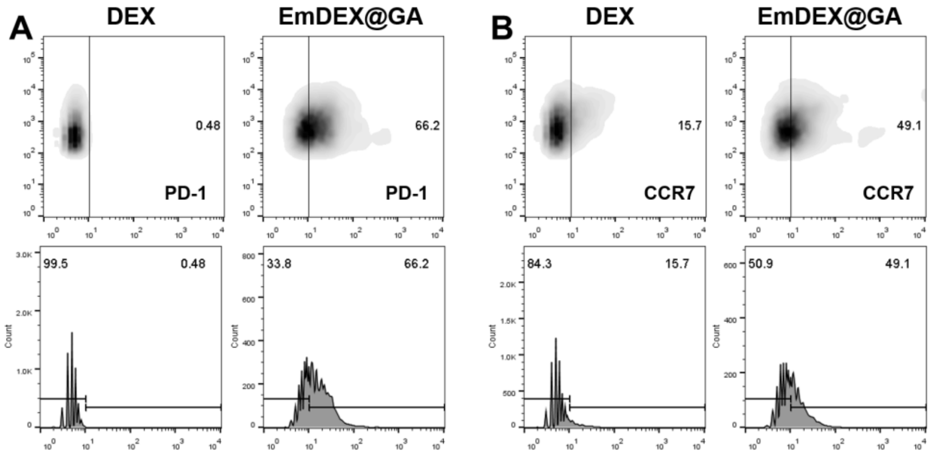

Figure S4. (A) PD-1 and (B) CCR7 expression percentages of EmDEX@GA compared with DEX.

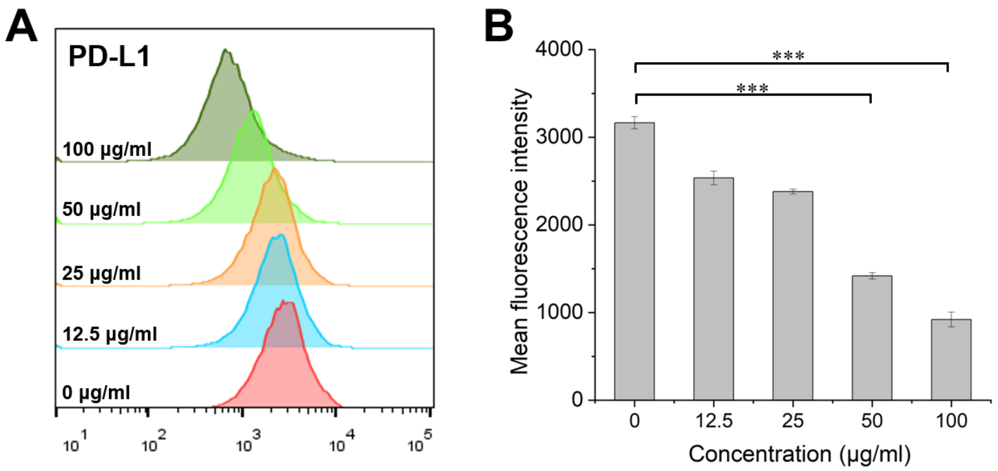

Figure S5. (A) PD-L1 detection on 4T1 cells treated with different concentrations of EmDEX@GA and its (B) MFI by flow cytometry ($n = 3$, ***$P < 0.001$).

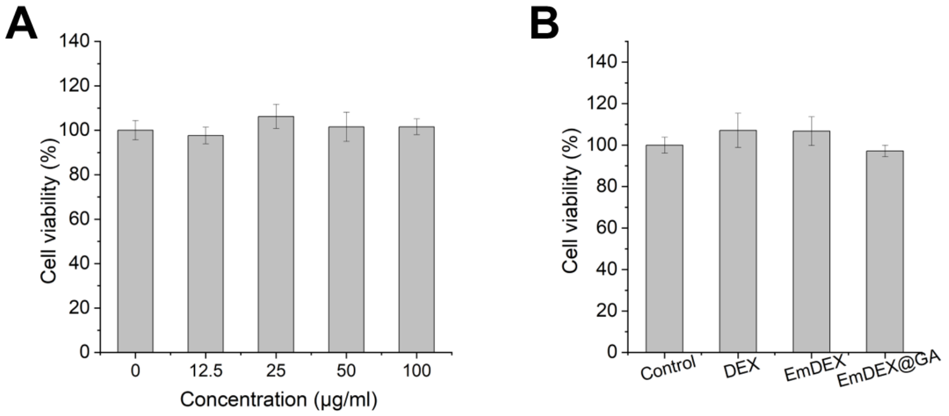

Figure S6. (A) Cell viability of 4T1 cells treated with different concentrations of EmDEX@GA and (B) different groups of exosomes.

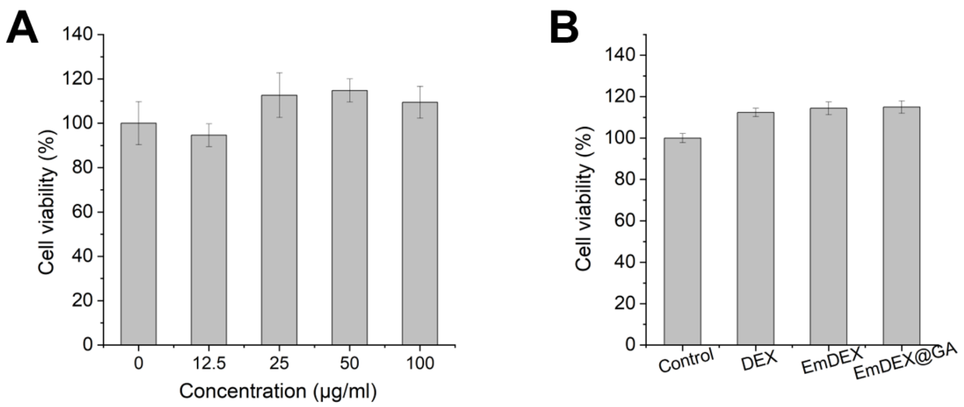

Figure S7. (A) Cell viability of DC2.4 cells treated with different concentrations of EmDEX@GA and (B) different groups of exosomes.

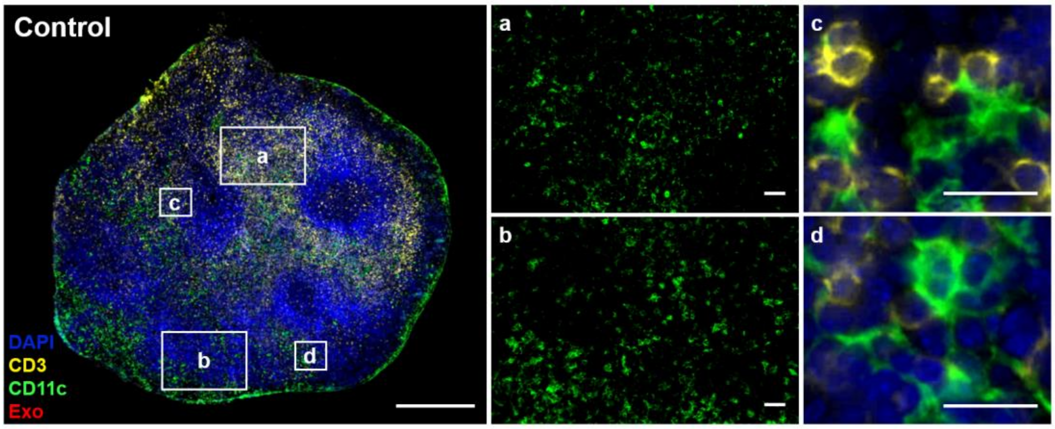

Figure S8. Representative immunofluorescence images of inguinal TDLN of mice in control group (blue: DAPI; yellow: CD3; green: CD11c; red: DiR-labeled exosomes; scale bar: 500 μm, 50 μm for a.b, 20 μm for c.d).

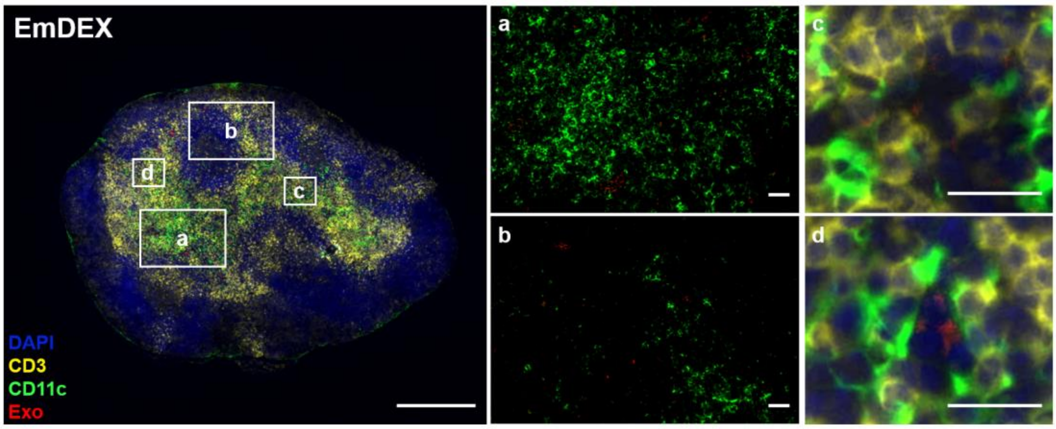

Figure S9. Representative immunofluorescence images of inguinal TDLN of mice in EmDEX group (blue: DAPI; yellow: CD3; green: CD11c; red: DiR-labeled exosomes; scale bar: 500 μm, 50 μm for a.b, 20 μm for c.d).

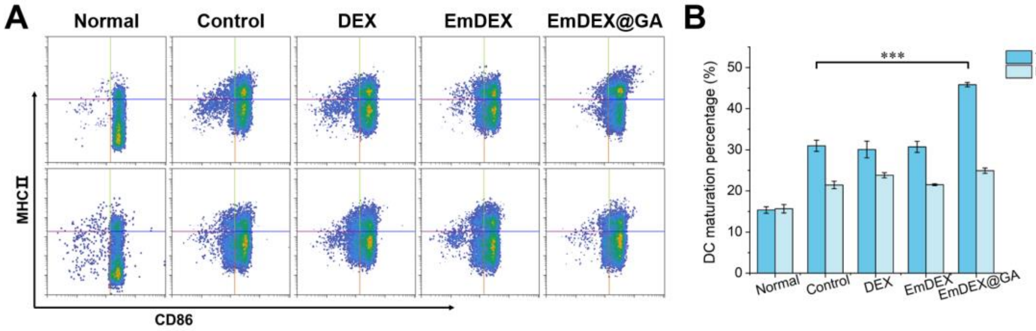

Figure S10. (A) Representative flow cytometric images and (B) quantitative analysis of the percentages of mature DCs in TDLN (right Ig.) and NDLN (left Ig.) ($n = 3$, $^{***}P < 0.001$).

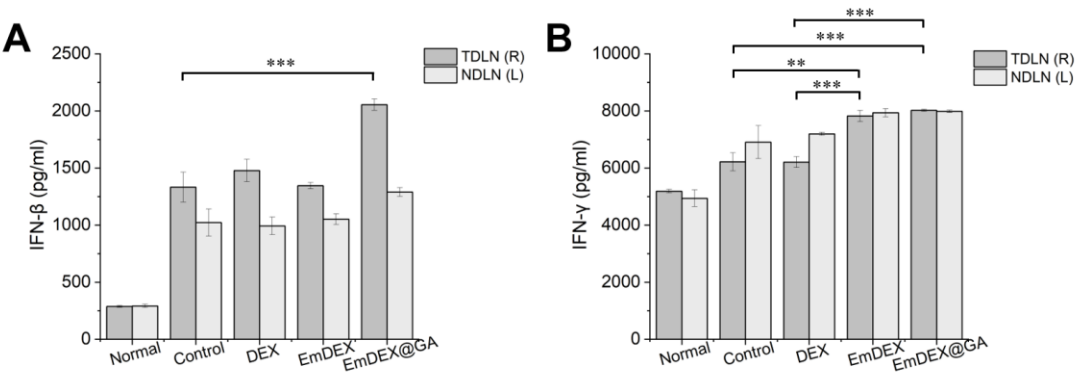

Figure S11. (A) The levels of IFN-β and (B) IFN-γ in TDLN (right lg.) and NDLN (left lg.) from mice in different groups (n = 3, **P < 0.01, ***P < 0.001).

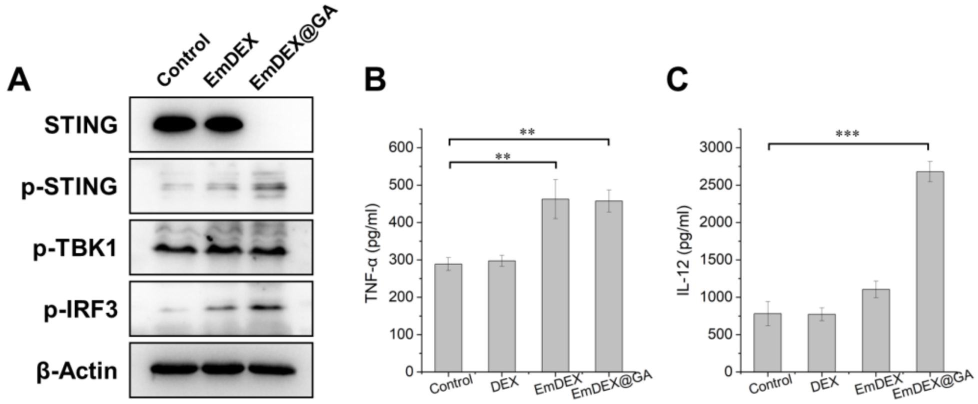

Figure S12. (A) WB analysis of the expression alterations associated with STING pathway. (B) The levels of TNF-α and (C) IL-12 in TDLN (right lg.) from mice in different groups (n = 3, **P < 0.01, ***P < 0.001).

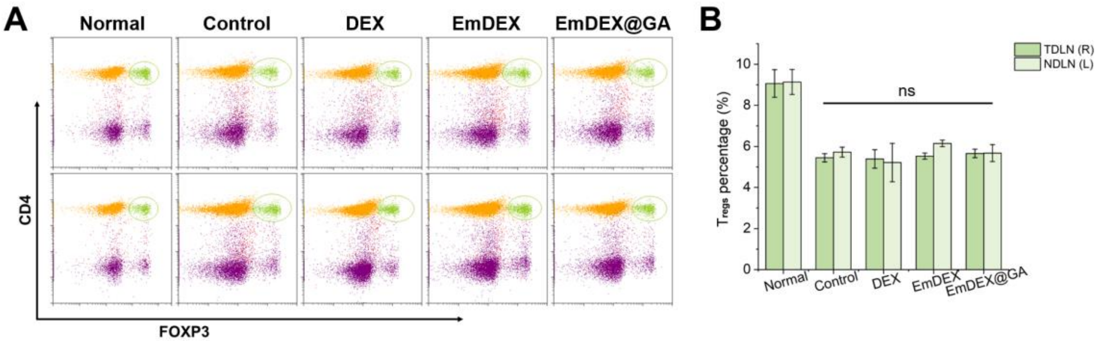

Figure S13. (A) Representative flow cytometric images and (B) quantitative analysis of the percentages of Tregs in TDLN (right Ig.) and NDLN (left Ig.) (n = 3, ns: not significant).

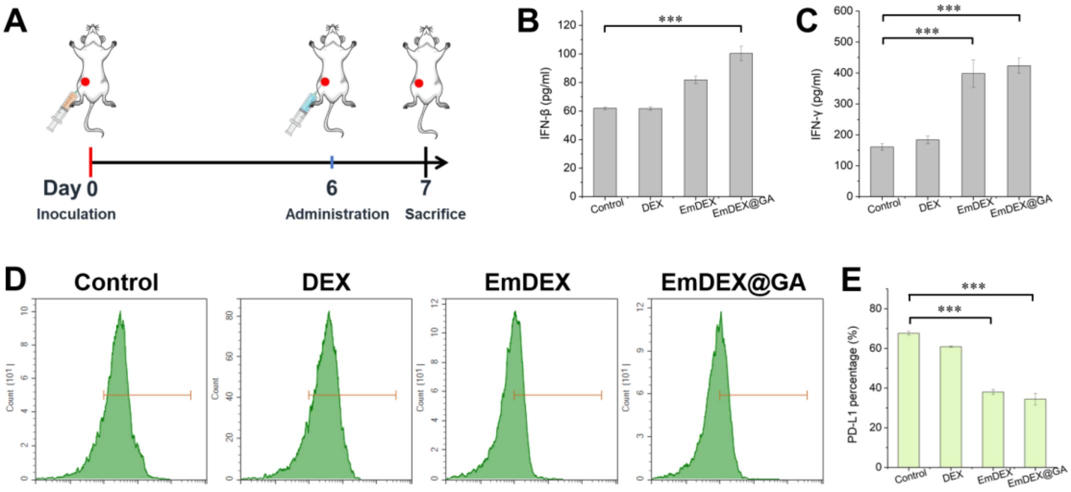

Figure S14. (A) Schematic diagram of the timeline in mice administration study. (B) The levels of IFN-β and (C) IFN-γ in tumor from mice in different groups (n = 3, ***P < 0.001). (D) PD-L1 expression in tumor and (E) quantitative analysis by flow cytometry (n = 3, ***P < 0.001).

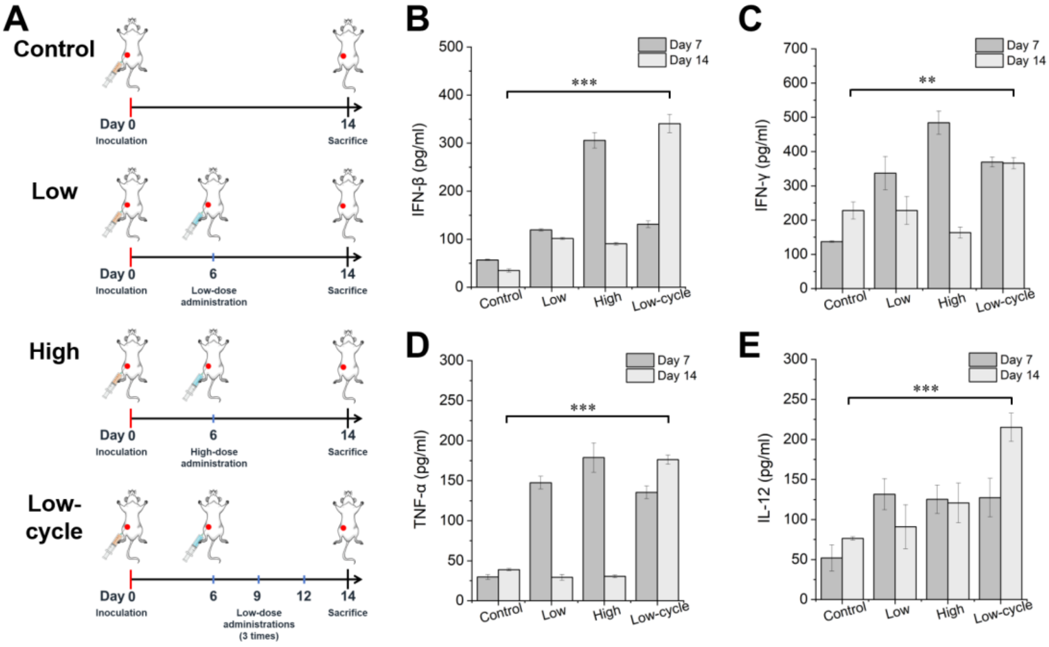

Figure S15. (A) Schematic diagram of the timelines in different groups of mice administration study. (B-E) The levels of (B) IFN-β, (C) IFN-γ, (D) TNF-α and (E) IL-12 in tumor from mice in different groups at day 7 and day 14 (n = 3, $^{**}P < 0.01$, $^{***}P < 0.001$).

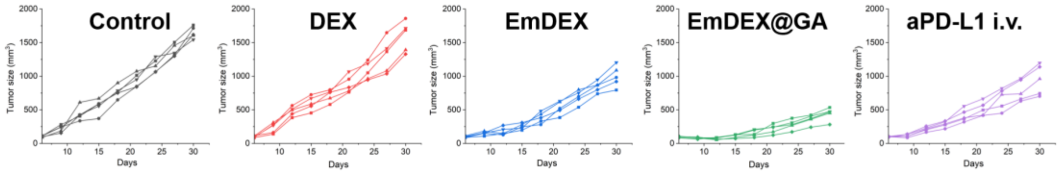

Figure S16. Tumor growth curves of mice in control, DEX, EmDEX, EmDEX@GA and aPD-L1 *i.v.* groups, respectively.

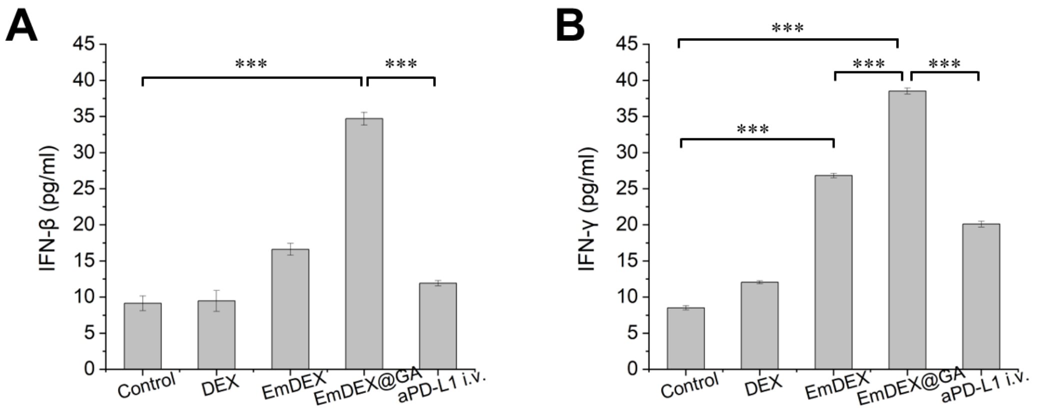

Figure S17. (A) The levels of IFN-β and (B) IFN-γ in tumor from mice in different groups (n = 3, ***P < 0.001).

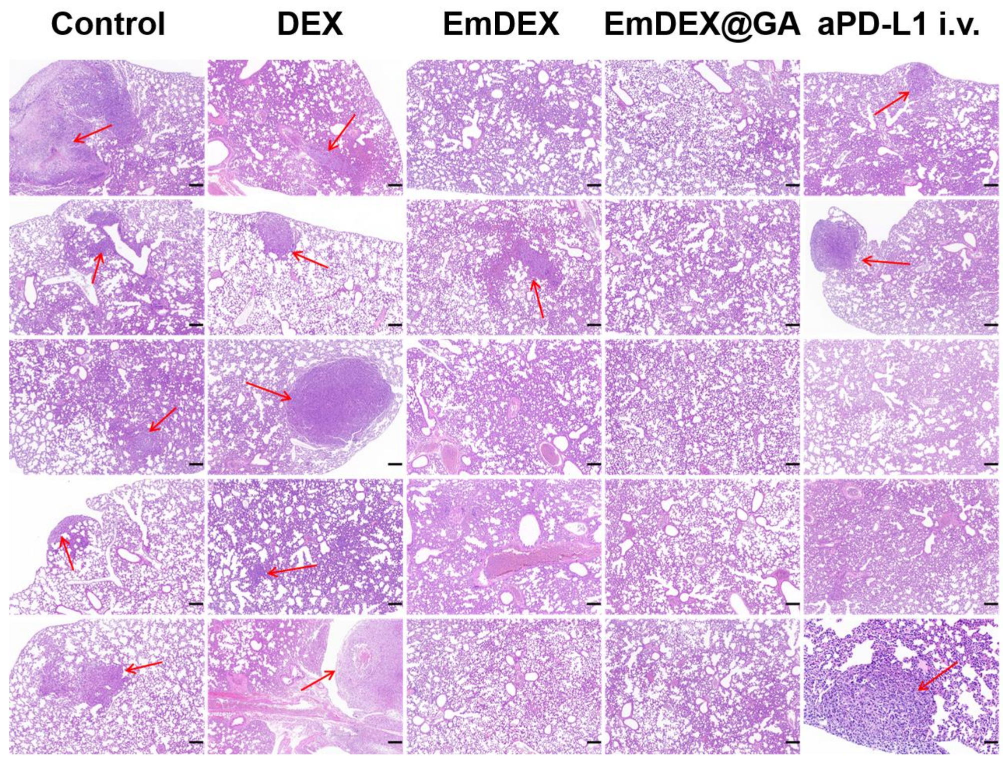

Figure S18. H&E staining images of lungs of mice in different groups. The location of metastasis was pointed by red arrow (scale bar: 200 μm).
